# Metabolic Regulation of a Bacterial Cell System with Emphasis on *Escherichia coli* Metabolism

**DOI:** 10.1155/2013/645983

**Published:** 2013-02-18

**Authors:** Kazuyuki Shimizu

**Affiliations:** ^1^Kyushu Institute of Technology, Fukuoka, Iizuka 820-8502, Japan; ^2^Institute of Advanced Bioscience, Keio University, Yamagata, Tsuruoka 997-0017, Japan

## Abstract

It is quite important to understand the overall metabolic regulation mechanism of bacterial cells such as *Escherichia coli* from both science (such as biochemistry) and engineering (such as metabolic engineering) points of view. Here, an attempt was made to clarify the overall metabolic regulation mechanism by focusing on the roles of global regulators which detect the culture or growth condition and manipulate a set of metabolic pathways by modulating the related gene expressions. For this, it was considered how the cell responds to a variety of culture environments such as carbon (catabolite regulation), nitrogen, and phosphate limitations, as well as the effects of oxygen level, pH (acid shock), temperature (heat shock), and nutrient starvation.

## 1. Introduction

Although living organisms may have been created somehow with the formation of a compartmentalized autocatalytic cycles with the appearance of ribonucleic acid-based or protein-based enzymes gaining complexity, evolved by adapting to the environment on earth, and improved in their effectiveness and robustness [1], it is still not certain that evolution can solve all the mystery of highly efficient, robust, and well-organized cell systems. It might be true that evolution has played some important roles for the improvement of cell's function and robustness to the changes in the environment, but this may not be all that can explain the cell's complexity with efficient function. In the living organisms, metabolic network, defined as the set and topology of metabolic biochemical reactions within a cell, plays an essential role for the cell to survive, where it is under organized control. In living organisms or cells, thousands of different biochemical reactions as well as transport processes are linked together to break down organic compounds to generate energy and to synthesize macromolecular compounds for cell synthesis. Note that the set of enzyme reactions is not static as illustrated in the biochemistry text book, but the set itself changes dynamically in response to the changes in the growth environment and the cell's state. Similarly, complex signaling networks interconvert signals or stimuli that are important for cellular function and interactions with the environment. This implies the transfer of information in signal transduction pathways and cascades designed to maximize efficiency and cellular responses. It may be of importance to understand the evolution of metabolism and signalings for understanding the adaptation processes of cellular life and the emergence of higher levels of organization [1]. However, here the time scale evolution will not be included unless otherwise stated, and a rather in-depth review is made on the metabolic regulation of a bacterial cell system.

The living organism must survive in response to the variety of environmental perturbations by maintaining the cell system by sensing external and/or internal state [2]. The main part of these functional responses concern the metabolic regulation. The enzymes which form the metabolic pathways are subject to multiple levels of regulation, where the transcriptional regulation may play the important role for metabolic regulation [3]. Although its relevance might have been overemphasized [4], it is important to understand the regulatory processes that govern the cellular metabolism. This is the key focus in the recent microbial systems biology, where genome-scale models for metabolic stoichiometry becomes popular [5, 6], together with the detailed topology of transcriptional regulatory networks that describe all known interactions between transcriptional factors and their target genes [3]. It should be noted that among possible topological network, only a subset is active at any given point in time and condition [7–10]. Although the transcriptional regulation is typically analyzed by measurements of mRNA abundance, the intracellular reaction rates or metabolic fluxes must be quantified in particular by ^13^C-based metabolic flux analysis to assess metabolic function in the network context [11–17]. It has been demonstrated the strong condition dependence of metabolic control with typically sparse networks of active transcriptional control that affect the flux distribution between different pathways in yeast [18]. 

Van Rijsewijk et al. [12] considered 81 transcription factors, including all known to directly or indirectly control central metabolic enzymes and 10 sigma- and anti-sigma factors in *E. coli*. Of the 81 transcription factors, 41 have one or more direct target gene that is involved in the central carbon metabolism [12]. In order to clarify the overall picture of the regulation, it is important to integrate different levels of omics data such as transcriptomics, proteomics, metabolomics, and fluxes, where advanced high throughput data are useful. Perhaps the most comprehensive data set containing those for *E. coli* grown at different growth rates in glucose-limited continuous cultures and upon deletion of 24 glycolysis and pentose phosphate pathway genes [19] may provide an unprecedented opportunity for computational analyses to extract biological insights [20]. The viability of living organism depends on the correct regulation of gene expressions, so that the appropriate proteins and enzymes must be produced in response to the environmental perturbation. Network analysis has been paid recent attention for investigating the transcriptional regulatory systems [21]. Babu et al. [22] considered to relate network topology to the systems' function in directing gene expression changes. In order to provide a comprehensive view of transcription factors (TFs) and their regulatory functions, RegulonDB is an invaluable resource in analyzing *E. coli* metabolic regulation [23]. Living organisms sense the changes in the environmental condition by detecting extracellular signals such as the concentrations of nutrients such as carbon, nitrogen, and phosphate sources, and the growth condition such as pH level, oxygen availability. These signals eventually feed into the transcriptional regulatory systems, which affect the physiological and morphological changes that enable organisms to adapt effectively for survival [24]. It has been highlighted that three quarters of *E. coli* TFs respond directly to external stimuli [25]. The 120 *E. coli* TFs may be classified into three categories depending on the original input signals such as external (58 TFs), internal (29 TFs), and hybrid (33 TFs).

**Figure 1:**
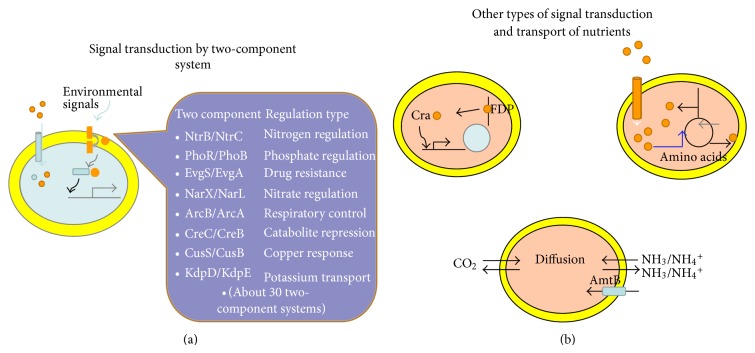
Transcription factors and their function.

The typical TFs in *E. coli *contains “two-headed molecules” which constitutes of a DNA-binding domain and an allosteric site to which metabolites bind noncovalently or to which enzymes covalently modify in order to modulate the regulatory activity of TF [24]. As will be mentioned later, two-component signal transduction system is considered to be the important means of detecting extracellular signals and transducing the signals into cytosol for metabolic regulation. These typically involve a phospho-relay from a transmembrane histidine protein kinase sensor to a target TF response regulator (Figure 1(a)). In the case of *E. coli*, 29 TFs show such regulation with 28 histidine protein kinase [26], where the genes encoding the two components are usually located within the same operon, enabling their coordinated expression, while some of the kinases and regulators are not adjacent on the chromosome, and it is not straightforward to link the partner in such a case [24]. Note that there might exist a cross-talk between noncognate sensors and regulators [27], which complicates the analysis of metabolic regulation.

As typically seen in Lac repressor, another method for sensing exogenous signals is by TF binding of transported small molecules, and the TFs regulate the enzymatic pathways that process these molecules [24]. In addition to exogenous signals, cell can recognize the cell's state by detecting the intracellular metabolites synthesized internally by cytosolic enzymes. The typical example is Cra (FruR) which binds a key intermediate such as F1, 6BP (FDP) and regulates the carbon flow (Figure 1(b)) as will be explained later in more detail. There exist, yet hybrid type of TFs, where they sense the metabolites that are transported from the culture environment or synthesized endogenously (Figure 1(b)). This can be typically seen in regulating amino acid synthetic pathways, possibly because it is preferable for the cell to import essential metabolites where they are freely available rather than expend energy on their production [24].

**Figure 2:**
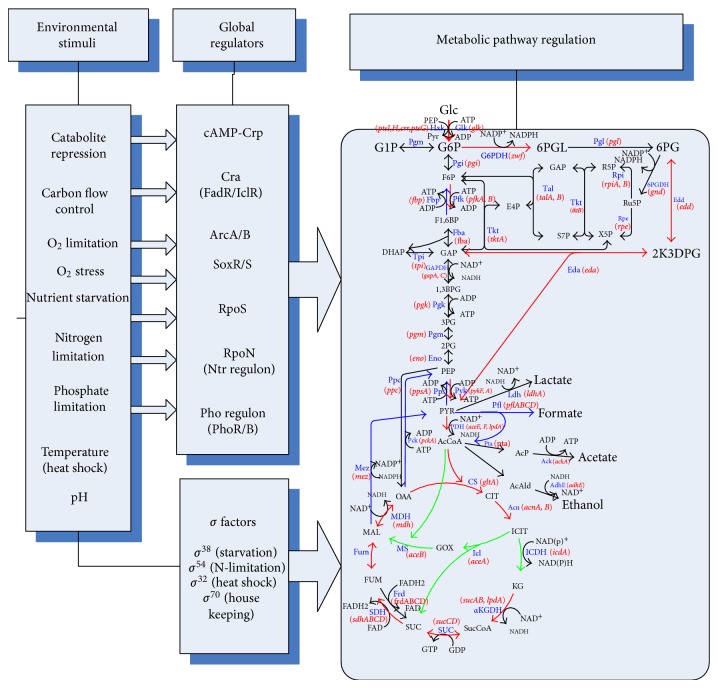
Overall metabolic regulation scheme.

The central metabolic pathways of a cell are controlled by a number of global regulators or transcription factors, depending on the culture condition as illustrated by Figure 2. Biological systems are known to be robust and adaptable to the culture environment. It became apparent that such robustness is inherent in the biochemical and genetic networks. Several genes that are necessary to respond to various environmental or nutritional changes require specific recognition by RNA polymerase associated with the alternative sigma factors. Here, we consider how the culture environment affects the global regulators as transcription factors, and how the metabolic pathway genes are regulated by the corresponding global regulators. 

## 2. Variety of Regulation Mechanisms

Living organisms such as bacterial cells have complex but efficient mechanisms to respond to the change in culture environment. This is achieved by the so-called global regulators, where they generally act at transcriptional level. The global transcriptional regulators are themselves regulated by posttranscriptional regulators. Thus, global regulation forms a cascade of regulations [28]. In relation to global regulators, sigma factors play also important roles, where they allow RNA polymerase to be recruited at specific DNA sequences in the promoter regions at which they initiate transcription. In *E. coli*, seven sigma factors have been found so far, and those play roles depending on the environmental stimuli (Table 1). In *Bacillus subtilis*, it has been known that multiple sigma factors control sporulation [29].

H-NS (histone-like nucleotide structuring protein) is another type of global transcriptional regulator, found in enterobacteria. It is a small DNA-associated protein that binds preferentially to a curved AT-rich DNA without showing sequence preferences [28]. H-NS regulates a variety of physiological pathways such as metabolism, fimbriae expression, virulence flagella synthesis, and proper function [28].

Other types of global regulators are signaling molecules such as cyclic-AMP (cAMP) and cyclic-di-GMP [30, 31]. As will be explained later in this paper, cAMP is synthesized from ATP by Cya (adenylate cyclase) at low glucose concentration with an increase in phosphorylated EIIA^Glc^ (EIIA^Glc^-P) involved in PTS, where EIIA^Glc^-P activates Cya activity. Note that cAMP binds to Crp (cAMP receptor protein), also known as CAP (catabolite activation protein), and cAMP-Crp complex becomes an activated transcription factor in relation to catabolite regulation. Note also that cAMP regulates not only catabolite regulation, but also flagellum synthesis, biofilm formation, quorum sensing, and nitrogen regulation [31–34].

As another level of regulation, small noncoding RNAs (sRNAs) play important roles in the posttranscriptional regulation [35]. The sRNAs are mainly involved in stress response regulation, pathogenesis, and virulence. A single sRNA can affect multiple targets, where sRNAs modify the translation or stability of the targets and chaperone. One such example is SgrS in *E. coli,* where it binds to the mRNA of *ptsG* gene, which encodes EIIBC^Glc^ for glucose uptake [36]. SgrS encodes a small protein SgrT, where SgrT is also involved in the inhibition of glucose uptake, and thus regulate *ptsG* activity [37]. Another sRNAs regulate other regulators, where such example is DsrA sRNA in *E. coli*, which regulates *σ* ^38^ expression [38]. Another group of sRNAs bind to proteins, where such example is CsrB in *E. coli* [39], and these sRNAs regulate the activity of CsrA global regulator as will be explained later for carbon storage regulation.

Another level of posttranscriptional regulation is the control of protein stability and folding carried out by ATP-dependent proteases and chaperones [28]. Such examples are the *E. coli* Lon ATP-dependent proteases that regulate flagella expression by degrading *σ* ^38^ as well as acid shock tolerance regulon by regulating the amount of GadE, where *gadE* is under *σ* ^38^ transcriptional control as will be explained later in this paper. As will be also explained, posttranslational control is mediated by the C1PXP ATP-dependent protease, which degrades *σ* ^38^.

## 3. Porin Proteins in the Outer Membrane and Their Regulation

The gram-negative bacteria such as *E. coli* have two concentric membranes surrounding the cytoplasm, where the space between these two membranes is called periplasm. The outer membrane and cytoplasmic membrane constitute a hydrophobic barrier against polar compounds. The outer membrane contains channel proteins, where the specific molecules can only move across these channels. In the outer membrane of *E. coli*, 10^8^ channels are formed by the porin proteins [40]. Porins are the outer membrane proteins that produce large, open but regulated water-filled pores that form substrate-specific, ion-selective, or nonspecific channels that allow the influx of small hydrophilic nutrient molecules and the efflux of waste products [41]. They also exclude many antibiotics and inhibitors that are large and lipophilic [42]. Porins including OmpC and OmpF of *E. coli* form stable trimers with a slight preference for cations over anions [43, 44]. The OmpC and OmpF are the most abundant porins present under typical growth condition representing up to 2% of the total cellular protein [45]. OmpF seems to have slightly larger channel than OmpC. OmpC and OmpF are the constitutive porins. Their relative abundance changes depending on such factors as osmolarity, temperature, and growth phase [46–48]. These porins serve for glucose to enter into the periplasm when glucose is present at a higher concentration than about 0.2 mM [49, 50]. It has been shown that the diffusion rate for glucose is found to be about twofold higher through OmpF than through OmpC [51]. Under glucose limitation, the outer membrane glycoporin LamB is induced [50], where this protein permeates several carbohydrates such as maltose, maltodextrins, and glucose [52]. It has been reported that about 70% of the total glucose import capacity of the cell is contributed by LamB [50]. Glucose transport by diffusion through porins of the outer membrane is a passive process [53]. 

**Figure 3:**
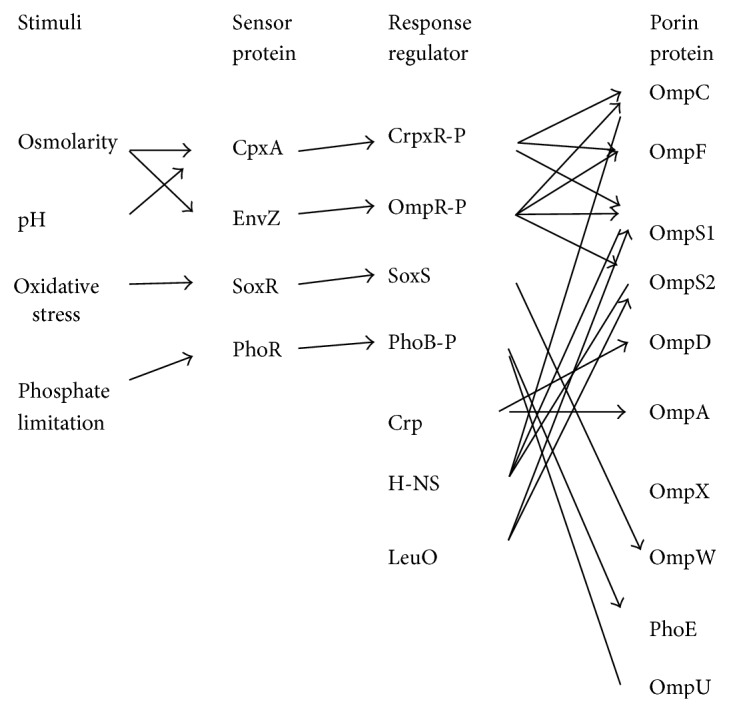
Outer membrane porin proteins and their regulations.

The porin genes are under control of two-component system such as EnvZ-OmpR system, where EnvZ is an inner membrane sensor kinase and OmpR is the cytoplasmic response regulator (Figure 3). In response to the environmental signals such as osmolarity, pH, temperature, nutrients, and toxins, EnvZ phosphorylates OmpR to form phosphorylated OmpR (OmpR-P), where OmpR-P increases its binding affinity for the promoter regions of porin genes such as *ompC* and *ompF* [41]. Note that acetyl phosphate can function as a phosphate donor for OmpR under certain condition. Note also that OmpR controls cellular processes such as chemotaxis and virulence as well [54]. In terms of virulence, abolition of porin production diminishes pathogenesis [41]. There are several other porins than OmpC and OmpF, such as OmpU and OmpT (*V. cholerae*), OmpH and OmpL (*Photobacterium*), OmpD (*S. typhimurium*), OmpS1 and OmpS2 (*S. enterica*), and OmpW (*S. enterica, E. coli*, and *V. cholerae*). Porin genes are also under control of other regulators other than EnvZ-OmpR such as CpxR (under extracytoplasmic stress) [54], PhoB (under phosphate limitation) [55], Lrp (under starvation) [56] Rob (for cationic peptides), MarA (under weak acids), SoxS (under oxidative stress) [57], CadC (at low pH) [58], Crp (under catabolite repression), Fnr (for anaerobiosis) [59], ToxR (for virulence) [60], H-NS, StpA, Ihf, Hu (for nucleotide proteins) [61], and LeuO (for stringent response) [41, 62].

CpxA and CpxR form a two-component system, where CpxA is the transmembrane sensor kinase, while CpxR is the response regulator. CpxA can be induced by a variety of stimuli such as higher pH (alkali), misfolded proteins and alterations in the membrane composition [63, 64]. Upon the activation of the kinase activity of CpxA, the phosphorylated CpxR (CpxR-P) plays roles as a transcriptional regulator and controls the expressions of *ompC *and *ompF* genes, and so forth [41].

PhoR and PhoB also form a two-component system, where phoR is the sensor kinase and detect a low concentration of phosphate or phosphate starvation and activate and phosphorylate PhoB [65] as will be explained later for phosphate regulation. The phosphorylated PhoB (PhoB-P) activates the transcription of *phoE* gene, where PhoE porin is induced under phosphate limitation [41]. Moreover, PhoB negatively regulates the OmpT, OmpU, and OmpA porins in *Vibrio cholera* [55].

Lrp is a global regulator which regulates mainly amino acid metabolism. Lrp activity is stimulated in minimal medium (which means low nutrient availability), while it is repressed in rich medium such as LB medium [66]. In minimal medium, Lrp negatively regulates *ompC* gene, while it positively regulates *ompF* gene.

MarA, SoxS, and Rob are the members of the AraC/XylS family of transcriptional regulators [67]. These three regulators diminish *ompF* expression [57]. SoxR and SoxS form a two-component system, where SoxR is a cytoplasmic sensor protein activated by oxidative stress and activates the SoxS regulator as will be explained later for oxidative stress regulation. MarA responds to weak acids like salicylic acid, and so forth [68] and certain antibiotics [69]. Rob may be a general regulator and might be stimulated by cationic peptides [70].

CadC is an inner membrane transcriptional activator that acts both as a signal sensor and as a transcriptional regulator, where it positively regulates the production of OmpC and OmpF at low pH [71, 72].

Crp plays an essential role for catabolite regulation as will be explained later, where Crp regulates *ompR-envZ* operon by binding directly to the promoter region [73]. The *ompA* gene in *E. coli* is positively regulated by Crp [74], while *ompX *is negatively regulated by Crp by means of CyaR, a small RNA (sRNA) [75]. In Typhimurium, *ompD* porin gene is activated by cAMP-Crp [59].

Fnr is a DNA-binding protein that senses oxygen level and regulates the metabolism under anaerobic condition together with ArcA/B regulator system as will be explained later for the metabolic regulation under oxygen-limited condition. Fnr positively regulates *ompD* gene expression under anaerobiosis by the posttranscriptional regulation [59].

ToxR is a transmembrane DNA-binding protein, and it is an important regulator of virulence gene expression in *V. cholera*. ToxR positively regulates *ompT* porin gene expression. Note that increased osmolarity enhances OmpT production and diminishes OmpU production, which is similar to that of OmpR on *ompC* and *ompF* in *E. coli*. Moreover, TorX represses *ompW* gene expression at high osmolarity in *V. cholerae* in the presence of glucose [76].

 Bacteria possess small nucleotide proteins such as H-NS, StpA, Ihf, and Hu with functional similarity to eukaryotic histones, which affect several porin genes [41]. H-NS is a master global regulator, which controls the expressions of several porin genes such as *ompC, ompF, ompS1*, and *ompS2*. H-NS represses *ompC* gene expression and diminishes the production of OmpF. StpA is a paralogue of H-NS and is an RNA chaperone. H-NS and StpA repress *ompS1* and *ompS2* gene expressions in* E. coli* and *S. Typhimurium.* [77, 78]. On the other hand, H-NS and StpA stimulate the production of the outer membrane maltoporin LamB through posttranscriptional control of the maltose regulon activator MalT [79].

Ihf is one of the most abundant sequence-specific DNA-binding proteins and is a global regulator. The Ihf protein negatively regulates *ompC* expression, and it is necessary for the negative osmoregulation of *ompF*. Ihf affects* ompC* and *ompF* in two distinct ways: directly by binding upstream to the promoter regions and indirectly by influencing the expression of EnvZ-OmpR [80].

LeuO is a LysR-type regulator that controls the expression of several genes in response to stress, virulence, and biofilm accumulation. The OmpS1 and OmpS2 quiescent porins are silenced by H-NS [78, 81], while LeuO acts as an antagonist of H-NS, thereby derepressing *ompS1* and *ompS2* gene expressions [41, 62].

Small untranslated regulatory RNAs, often referred to as noncoding RNA, also affect porin regulation. MicF is one such example. In general, they inhibit translation of the transcripts by direct RNA-RNA interaction [41]. The sRNAs have been found to play diverse physiological roles in response to stress, metabolic regulation, control of bacterial envelope composition, and bacterial virulence [38, 82–84]. It has been shown that enterobacteria use many sRNAs such as MicC, MicA, InvR, RybB, CyaR, IpeX, and RseX to fine-tune the outer membrane composition at the posttranscriptional level [84].

## 4. Transport of Carbohydrates and Carbon Catabolite Regulation

### 4.1. Transport of Substrate Molecules and PTS

**Figure 4:**
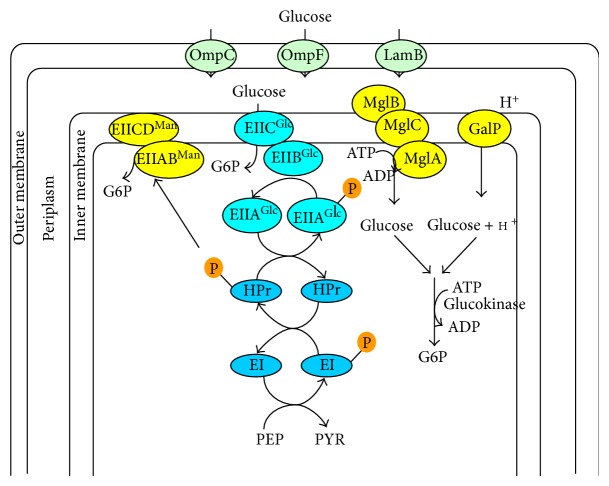
Outer and inner membrane and periplasm and glucose transport by PTS and non-PTS.

The first step in the metabolism of carbohydrates is the transport of these molecules into the cell (Figure 4). In bacteria, various carbohydrates can be taken up by several mechanisms [85]. Primary transport of sugars is driven by ATP, while secondary transport is driven by the electrochemical gradients of the translocated molecules across the membrane [86], where the secondary transport systems contain the symporters which cotransport two or more molecules, uniporters that transport single molecule, and antiporters that countertransport two or more molecules. Sugar symporters usually couple the uphill movement of the sugar to the downhill movement of proton (or sodium ion). Namely, the electrochemical proton (or sodium ion) gradient drives the accumulation of glucose [85]. Sugar uptake by group translocation is unique for bacteria and is involved in the phosphotransferase system (PTS) (Figure 4).

Once glucose was transported inside periplasm, it can be internalized into the cytoplasm by the phosphotransferase system (PTS). It may be considered that the glucose concentration in the periplasm is low due to active transport systems in the cytoplasmic membrane [53]. Once inside the periplasm, glucose can be transported into cytosol by PTS, where PTS is widespread in bacteria and absent in archaea and eukaryotic organisms [87]. PTS is composed of the soluble and nonsugar-specific components Enzyme I (EI) encoded by *ptsI* and the phosphohistidine carrier protein (HPr) encoded by *ptsH*, where they transfer phosphoryl group from PEP to the sugar-specific enzyme IIA and IIB. Another component of PTS, is the enzyme IIC (in some cases also IID) which is an integral membrane protein permease that recognizes and transports the sugar molecules, where it is phosphorylated by EIIB. There have been reported to exist 21 different enzyme II complexes in *E. coli*, that are involved in the transport of about 20 different carbohydrates [88]. In *E. coli*, EII^Glc^ and EII^Man^ are involved in the transport of glucose. The EII^Glc^ is composed of the soluble EIIA^Glc^ encoded by *crr* and of the integral membrane permease EIICB^Glc^ encoded by *ptsG.* The EII^Man^ complex is composed of the EIIAB^Man^ homodimer enzyme and the integral membrane permease EIICD^Man^ (Figure 4), where these proteins are encoded in the *manXYZ* operon [53]. In addition to mannose, these proteins can also transport glucose, fructose, N-acetylglucosamines, and glucosamine with similar efficiency [89]. In a wild-type strain growing on glucose, *ptsG* is induced, while *manXYZ* operon is repressed. In *ptsG* mutant, the glucose can be transported by EII^Man^ complex, and the cell can grow with less growth rate than the wild-type strain [90]. When the extracellular glucose concentration is less than about 1 *μ*M, or it is more than about 2 g/L for *pts* mutants, this can be also utilized [91]. The induction of these genes is caused by the intracellular galactose that functions as an autoinducer of the system [92]. One of the genes induced under glucose limitation is *galP*, that codes for the low-affinity galactose: H^+^ symporter GalP (Figure 4). 

The genes in the *mglABC* operon encode an ATP-binding protein, a galactose/glucose periplasmic binding protein, and an integral membrane transporter protein, respectively, forming Mgl system for the galactose/glucose (methyl galactoside) import [53]. This high-affinity porter belongs to the ATP-binding cassette (ABC) superfamily of the primary active class of transporters [53]. When extracellular glucose concentration is very low, the Mgl system together with LamB attains high-affinity glucose transport [53]. The glucose molecule transported either by GalP or Mgl systems must be phosphorylated by Glk encoded by *glk* from ATP to become G6P (Figure 4) [93]. 

Note that PTS seems to be quite efficient as it consumes one mole of PEP for each internalized and phosphorylated glucose, where one mole of PEP is equivalent to one mole of ATP, since the conversion of PEP to PYR by Pyk would yield one mole of ATP by substrate-level phosphorylation. The high-affinity Mgl-glucokinase system is energetically the most expensive, as it consumes two moles of ATP per glucose. The GalP-glucokinase system requires one mol of H^+^ that is internalized into the cytoplasm and one mol of ATP (Figure 4).

### 4.2. Carbon Catabolite Regulation

Among the culture environment, carbon sources are by far important for the cell from the point of view of energy generation and biosynthesis. Most living organisms including bacteria can use various compounds as carbon sources, where these can be either cometabolized or selectively used with preference for the specific carbon sources among available carbon sources. One typical example of selective carbon-source usage is the diauxie phenomenon observed in *Escherichia coli *and others when a mixture of glucose and other carbon sources such as lactose was used as a carbon source, where this phenomenon was first observed by Monod [94]. Subsequent investigation on this phenomenon has revealed that selective-carbon source utilization is common and that glucose is the preferred carbon source in many organisms. Moreover, the presence of glucose often prevents the use of other carbon sources. This preference of glucose over other carbon sources has been named as glucose repression, or more generally carbon catabolite repression (CCR) [95]. CCR is observed in most heterotrophic bacteria which include facultatively autotrophic bacteria that repress the genes for CO_2_ fixation in the presence of organic carbon source [96]. Some pathogenic bacteria such as *Chlamydia trachomatis* and *Mycoplasma pneumonia* seem to lack CCR, where these are adapted to nutrient-rich host environments [97, 98]. Another phenomenon can be seen in *Corynebacterium glutamicum*, where coassimilation of glucose and other carbon sources is made, but it is highly regulated [99, 100]. It is of interest that for some bacteria such as *Streptococcus thermophilus*, *Bifidobacterium longum*, and *Pseudomonas aeruginosa*, glucose is not a primary carbon source, and the genes for glucose utilization are repressed when preferred carbon sources are available, where this phenomenon is called as reverse CCR [101–103]. CCR is one of the most important regulatory phenomena in many bacteria [104–106]. CCR is important for the cells to compete with other organisms in nature, where it is crucial to select a preferred carbon source in order to improve the growth rate, which then results in survival as compared to other competing organisms. Moreover, CCR has a crucial role in the expression of virulence genes, which often enable the organism to access new sources of nutrients. The ability to select the appropriate carbon source that allows fastest growth may be the driving force for the evolution of CCR [107].

**Figure 5:**
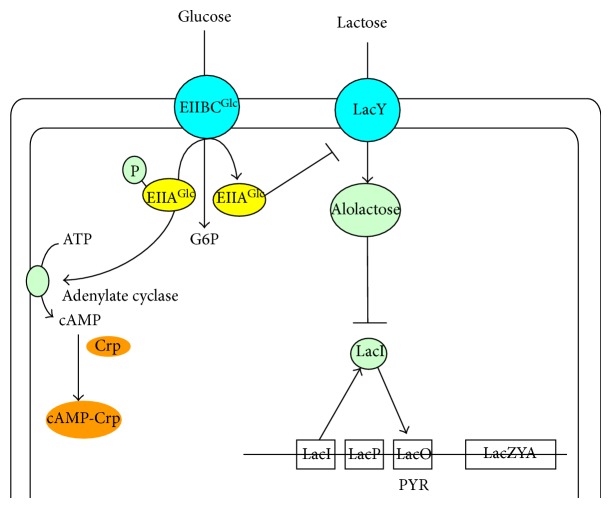
Inducer exclusion and the activation of adenylate cyclase in the glucose-lactose system.

The *E. coli lac* operon is only expressed if allolactose (a lactose isomer formed by *β*-galactosidase) binds and inactivates the* lac* repressor. Lactose cannot be transported into the cell in the presence of glucose, because the lactose permease, LacY is inactive in the presence of glucose [108]. As shown in Figure 5, phosphorylated EIIA^Glc^ is dominant when glucose is absent and does not interact with LacY, whereas unphosphorylated EIIA^Glc^ can bind and inactivates LacY when glucose is present [109, 110]. Note that this only occurs if lactose is present [111]. The same mechanism may be seen for the transport of other secondary carbon sources such as maltose, melibiose, raffinose, and galactose [112, 113]. 

Inducer exclusion has also been reported for Gram positive bacteria, and HPr is the major player in these organisms. In *Lactobacillus brevis*, HPr(Ser-P) is formed when glucose is present and binds and inactivate permease [114]. By contrast, the lactose permease of *S. thermophilus* is controlled by HPr-(His-P-) dependent phosphorylation. In the absence of glucose, HPr (His-P) can phosphorylate PTS-like domain, thereby activating the permease for lactose transport [115]. When glucose is present, HPr becomes phosphorylated on Ser46 and can no longer activate the lactose permease [116].

**Figure 6:**
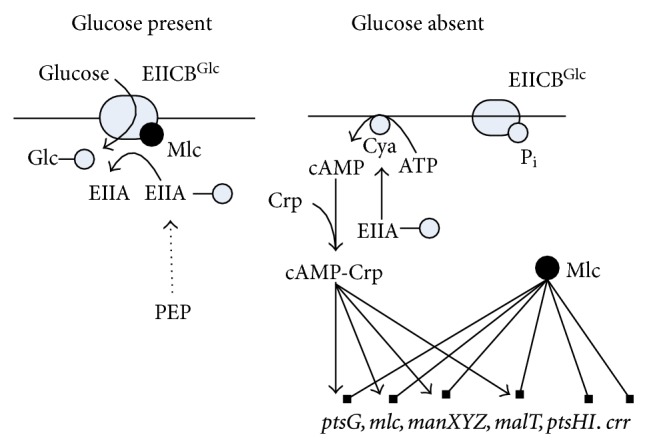
The multiple regulations by Mlc and cAMP-Crp.

The central players in carbon catabolite regulation in *E. coli* are the transcriptional activator Crp (cyclic AMP (cAMP) receptor protein; also called as catabolite gene-activator protein (CAP)), the signal metabolite cAMP, adenylate cyclase (Cya), and the PTSs, where these systems are involved in both transport and phosphorylation of carbohydrates. The PTS in *E. coli *consists of two common cytoplasmic proteins, EI (enzyme I) encoded by *ptsI* and HPr (histidine-phosphorylatable protein) encoded by *ptsH*, as well as carbohydrate-specific EII (enzyme II) complexes. The glucose-specific PTS in *E. coli* consists of the cytoplasmic protein EIIA^Glc^ encoded by *crr* and the membrane-bound protein EIICB^Glc^ encoded by *ptsG*, which transport and concomitantly phosphorylate glucose as explained before. The phosphoryl groups are transferred from PEP via successive phosphorelay reactions in turn by EI, HPr, EIIA^Glc^ and EIICB^Glc^ to glucose. The cAMP-Crp complex and the repressor Mlc are involved in the regulation of *ptsG* gene and *pts* operon expressions. It has been demonstrated that unphosphorylated EIICB^Glc^ can relieve the expression of *ptsG* gene expression by sequestering Mlc from its binding sites through a direct protein-protein interaction in response to glucose concentration. In contrast to Mlc, where it represses the expressions of *ptsG*, *ptsHI,* and *crr* [117], cAMP-Crp complex activates *ptsG* gene expression [118] (Figure 6). Since intracellular cAMP levels are low during growth on glucose, these two antagonistic regulatory mechanisms guarantee a precise adjustments of *ptsG* expression levels under various conditions [119] (Figure 6). It should be noted that unphosphorylated EIIA^Glc^ inhibits the uptake of other non-PTS carbohydrates by the so-called inducer exclusion [120], while phosphorylated EIIA^Glc^ (EIIA^Glc^-P) activates adenylate cyclase (Cya), which generates cAMP from ATP and leads to an increase in the intracellular cAMP level [121] (Figure 6). In the absence of glucose, Mlc binds to the upstream of *ptsG* gene and prevents its transcription. If glucose is present in the medium, the amount of unphosphorylated EIICB^Glc^ increases due to the phosphate transfer to glucose. In this situation, Mlc binds to EIICB^Glc^, and thus it does not bind to the operator region of *pts* genes [119, 122, 123]. Note that if the concentration ratio between PEP and PYR (PEP/PYR) is high, EIIA^Glc^ is predominantly phosphorylated, whereas if this ratio is low, then EIIA^Glc^ is predominantly dephosphorylated [124, 125]. EIIA^Glc^ is preferentially dephosphorylated when *E. coli* cells grow rapidly with glucose as a carbon source [124, 125]. Note also that cAMP levels are low during growth with non-PTS carbohydrates such as lactose, where PEP/PYR ratio is the key factor that controls phosphorylation of EIIA^Glc^, which explains dephosphorylation of EIIA^Glc^, resulting in low cAMP pool [124, 125]. As stated above, inducer exclusion is the dominant factor for the glucose-lactose diauxie [109, 126]. The roles of cAMP-Crp is then to express *lac* operon, and it is involved in CCR by activating the expression of *ptsG* and EIICB domain of the glucose-specific PTS, and therefore the transport of glucose [127].

### 4.3. Carbohydrate Uptake by Various PTS and without PTS in *E. coli*

**Figure 7:**
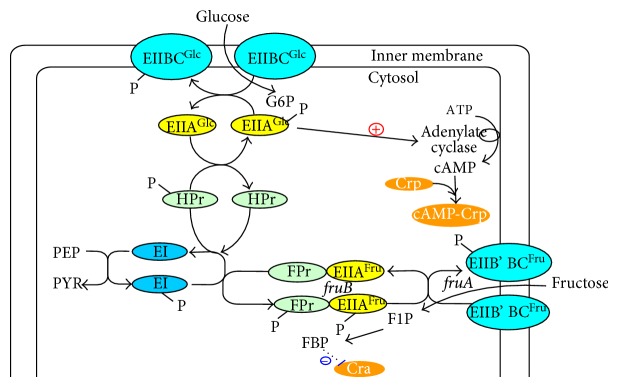
Glucose PTS and fructose PTS.

As stated above, PTS is a transport system that catalyzes the uptake of a variety of carbohydrates and their conversion into their respective phosphoesters during transport [128]. PTS is composed of EI, HPr, and E II, where those accept phosphoryl group from a donor and transfer to an acceptor, thus cycling between the phosphorylated and unphosphorylated states [128]. EI and HPr are common to all PTS carbohydrates, while EII is carbohydrate specific. Thus, bacteria usually contain many different E IIs. Each E II complex consists of one or two hydrophobic integral membrane domains (C and D) and two hydrophilic domains (A and B), which together are responsible for the transport of the carbohydrate across the membrane as well as its phosphorylation. *E. coli *contains different EII complexes, where EII complexes are formed either by distinct proteins that contain EI and/or HPr domains exist [128]. A prominent example for the latter is FPr, which consists of HPr and EIIA domain and mediates the phosphotransfer in the uptake of fructose by *E. coli*. As shown in Figure 7, fructose can be transferred and phosphorylated by the fructose PTS (EIIBC^Fru^) or ATP-dependent mannofruct kinase Mak [129]. The EIIBC^Fru^ encoded by *fruA *phosphorylate fructose concomitantly with transport to fructose 1-phosphate, which is further converted to FDP by an ATP-dependent Pfk [130]. 

**Figure 8:**
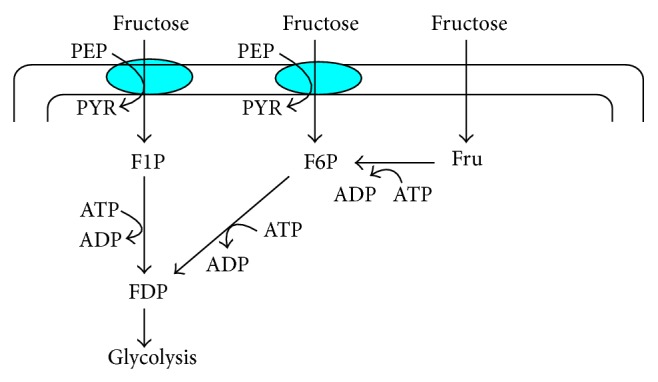
Fructose uptake pathways.

There are three pathways for the utilization of fructose as shown in Figure 8 [131]. In the primary pathway, fructose (Fru) is transported via the membrane-spanning protein FruA and concomitantly phosphorylated by a PEP: D-fructose 1-phosphotransferase (fructose PTS) system (ATP: D-fructose 1-phosphotransferase, EC 2.7.1.3), which is induced by D-fructose and enter the cell as D-fructose 1-phosphate (F1P), where this process is affected by the transfer of a phosphoryl moiety from PEP to the hexose by the concerted action of two cytoplasmic proteins: EI of PTS and a membrane-associated diphosphoryl protein (DTP). F1P is then converted to fructose 1,6-diphosphate (FDP) by ATP and by the inducible enzyme D-fructose-1-phosphate kinase (F1PK) (ATP: D-fructose-1-1phosphate 6-phosphotransferase). 

In the second pathway, fructose enters the cell via a membrane-spanning proteins that have a general ability to recognize sugars possessing the 3,4,5-D-arabino-hexose configuration which include the permeases for mannose (ManXYZ), glucitol (SrlA), and mannitol (MtlA) [131] D-fructose is converted to F6P by a specific sucrose-induced D-fructokinase (ATP: D-fructose 6-phosphotransferase, EC 2.7.1.4), and then converted to FDP by Pfk of the EMP pathway.

In the 3rd pathway, fructose enters the cell by diffusion, using an isoform of the glucose transporter PtsG. Since this mode of entry does not involve the PTS, the free fructose has to be phosphorylated by ATP to become F6P.

**Figure 9:**
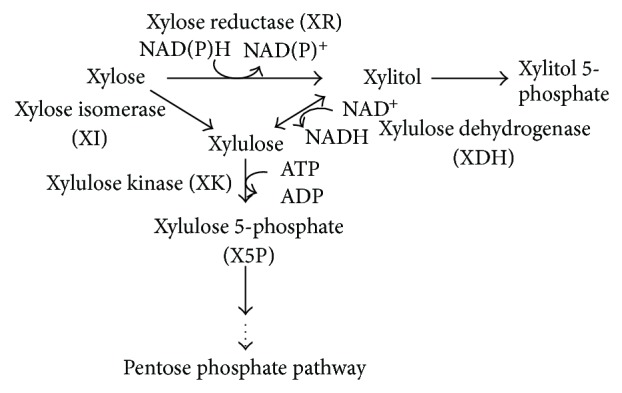
Xylose uptake pathways.

D-xylose is converted to D-xylulose by xylose isomerase (D-xylose ketoisomerase, EC 5.3.1.5) (Figure 9). D-Xylulose is subsequently phosphorylated by xylulokinase (ATP: D-xylulose 5-phosphotransferase, EC 2.7.1.17) to form D-xylulose 5-phosphate (X5P). Under anaerobic condition, xylulose reductase (XR) is induced, and xylitol and xylitol 5-phosphate are produced, where they may inhibit the cell growth.

**Figure 10:**
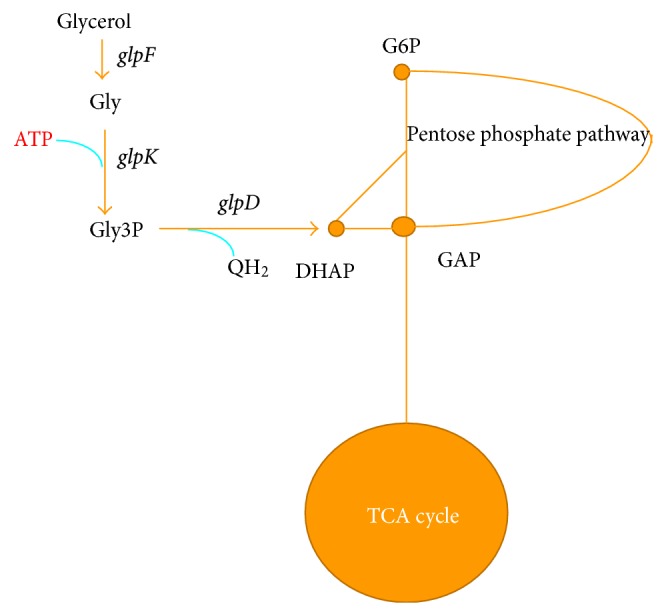
Glycerol uptake pathways.

Glycerol is oxidized to dihydroxyacetone by a glycerol dehydrogenase (glycerol: NAD oxidoreductase, EC 1.1.1.6). Dihydroxyacetone is then phosphorylated by a kinase using ATP (Figure 10). Another pathway for glycerol utilization is that glycerol is phosphorylated by glycerol kinase (ATP: glycerol phosphotransferase, EC 2.7.1.30) to form L-glycerol 3-phosphate, which then is converted to GAP.

### 4.4. CCR in Other Bacteria Than *E. coli*

The key players in CCR in *Bacillus subtilis* are the pleiotropic transcription factor CcpA (catabolite control protein A), the Hpr protein of the PTS, the bifunctional HPr kinase/phosphorylase (HPrK) and the glycolytic intermediates such as FDP and G6P [132–134]. Unlike *E. coli*, HPr phosphorylation plays an important role, where phosphorylated HPr serves as the effector for the dimeric CcpA, which controls the expressions of CCR genes [107]. The phosphorylation of HPr is catalysed by HPrK, that binds ATP, and its activity is triggered by the availability of FDP as an indicator of high glycolytic activity [135–137]. By contrast, phosphorylase activity prevails under nutrient limitation, and the activation is stimulated by the inorganic phosphate in the cell [137, 138]. Under nutrient rich condition, HPrK acts as a kinase and phosphorylates HPr, and the cofactor for CcpA is formed. The interaction between CcpA and the phosphorylated HPr is enhanced by FDP and G6P [139, 140]. 

With the exception of the mycoplasmas, a Firmicutes also use HPr, HPrK, and CcpA for CCR [132]. CcpA in lactic acid bacteria such as *Lactococcus lactis *represses not only genes of carbon metabolism, but also controls metabolic pathway genes such as glycolysis and lactic acid formation pathway genes [107]. 

In *streptomyces coelicolor* and the related species, glucose kinase is the key player of CCR, where it is independent of the PTS [141]. *Corynebacterium glutamicum* is important in the industrial production of amino acids, where it prefers to use multiple carbon sources simultaneously. Diauxic growth is observed for the case of using a mixture of glutamate or ethanol and glucose, where the repressor protein RamB is activated when glucose is present and binds to the promoter regions of the genes involved in acetate and ethanol catabolism [142, 143]. The *ramB* expression is regulated by the feedback of RamB and RamA, where RamA is activated when acetate is present. The 13 two-component systems have been found so far, and the role of five has been elucidated recently [144].

*Pseudomona putida *can assimilate various aromatic and aliphatic hydrocarbons, where it has been reported that the use of hydrocarbons is represses by succinate, and this seems to be a general feature of CCR in this organism [103, 145]. Under CCR, the translation of operon-specific regulators is inhibited by the binding of an RNA-binding protein Crc to mRNAs of the regulator transcript, and thus CCR seems to be governed by an RNA-binding protein at the level of posttranscriptional control rather than by a DNA-binding transcriptional regulator [146, 147].

CCR is crucial for the expression of virulence genes and for pathogenicity in many pathogenic bacteria. Note that the primary aim of pathogenic bacteria is to gain access to nutrients rather than to cause damage to the host and that the expression of virulent genes is linked to the nutrient supply of the bacteria [107].

In many Firmicutes, the mutants devoid of the HPr kinase grow significantly slower than wild-type cells. It is, therefore, suggested that HPr kinase, which generates the cofactor for CcpA, might be a suitable drug target, where the compound that inhibits the kinase activity of HPr has been identified, and this compound inhibits the growth of *B. subtilis* but not of *E. coli*, where *E. coli* does not contain HPr kinase [107].

Crp and cAMP are essential for the expression of virulence genes in enteric bacteria, and therefore, the corresponding *crp* and *cya* mutant strains of *S. enterica* and *Y. enterocolitica* can be used as live vaccines in mice and pigs [148–150].

### 4.5. Carbon Storage Regulation

**Figure 11:**
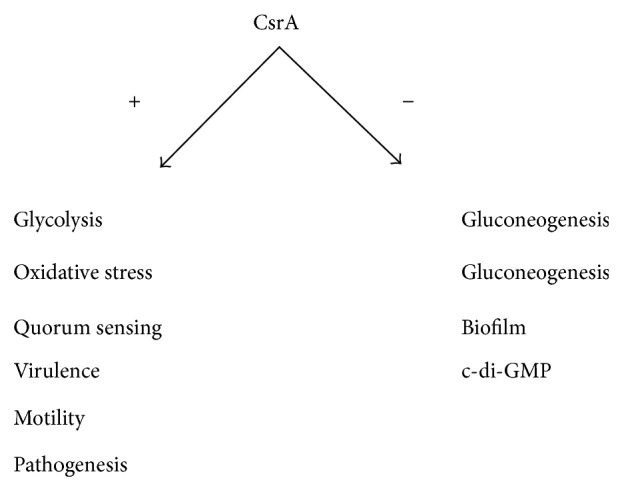
Regulation of Csr.

The carbon storage regulator (Csr) system influences a variety of physiological processes such as central carbon metabolism, biofilm formation, motility, peptide uptake, virulence and pathogenesis, quorum sensing, and oxidative stress response [28, 151–153] (Figure 11). Csr is controlled by the RNA-binding protein CsrA, a posttranscriptional global regulator that regulates mRNA stability and translation [154–156]. CsrA binds to the 5′ untranslated region of its target mRNAs, often in the region spanning the Shine-Dalgarno (SD) site [157]. CsrA is regulated by two sRNAs called CsrB and CsrC in *E. coli* [158–160]. These sRNAs are composed of multiple CsrA-binding sites that bind and sequester CsrA [39].

**Figure 12:**
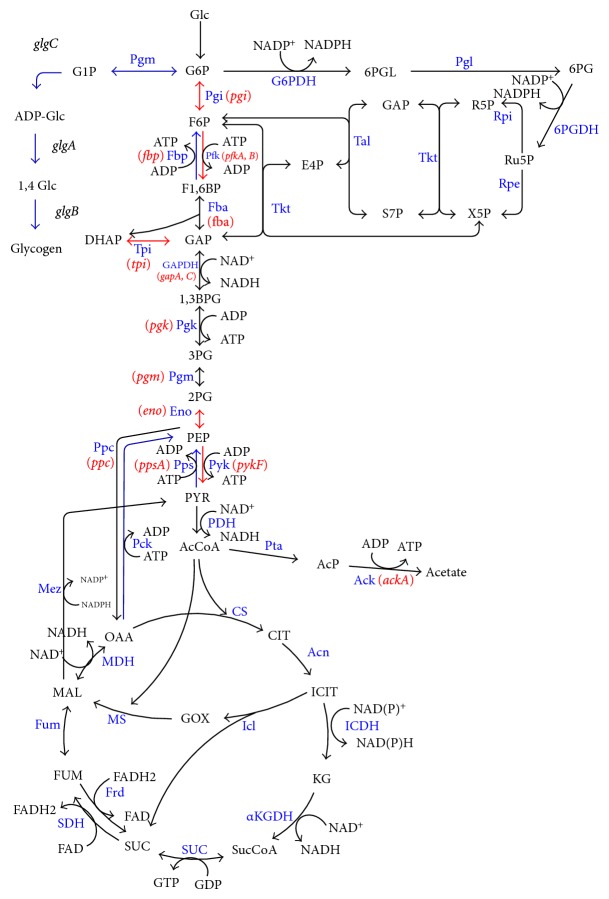
Pathway regulation by CsrA.

CsrA is a global regulator and regulates a variety of pathways as stated above, where the central carbon metabolism is of practical interest among them. CsrA negatively regulates glycogen accumulation by regulating the expressions of *glgCAP* operon and *glgB* of *glgBX* operon [28, 161]. As illustrated in Figure 12, CsrA regulates central carbon metabolism and glycogenesis such that glycogen synthesis pathway genes such as *pgm, glgC, glgA*, and *glgB*, as well as gluconeogenic genes such as *fbp, ppsA*, and *pckA* genes are repressed, while glycolysis genes such as *pgi, pfkA* (but not *pfkB*), *tpiA, eno,* and *pykF* genes are activated [28, 162]. It has been shown that phenylalanine production could be enhanced by manipulation of Csr [163]. More recently, it was shown that biofuel production could be enhanced by manipulating (enhancing) CsrB in *E. coli *[162].

### 4.6. Carbon Flow Control in *E. coli*

**Figure 13:**
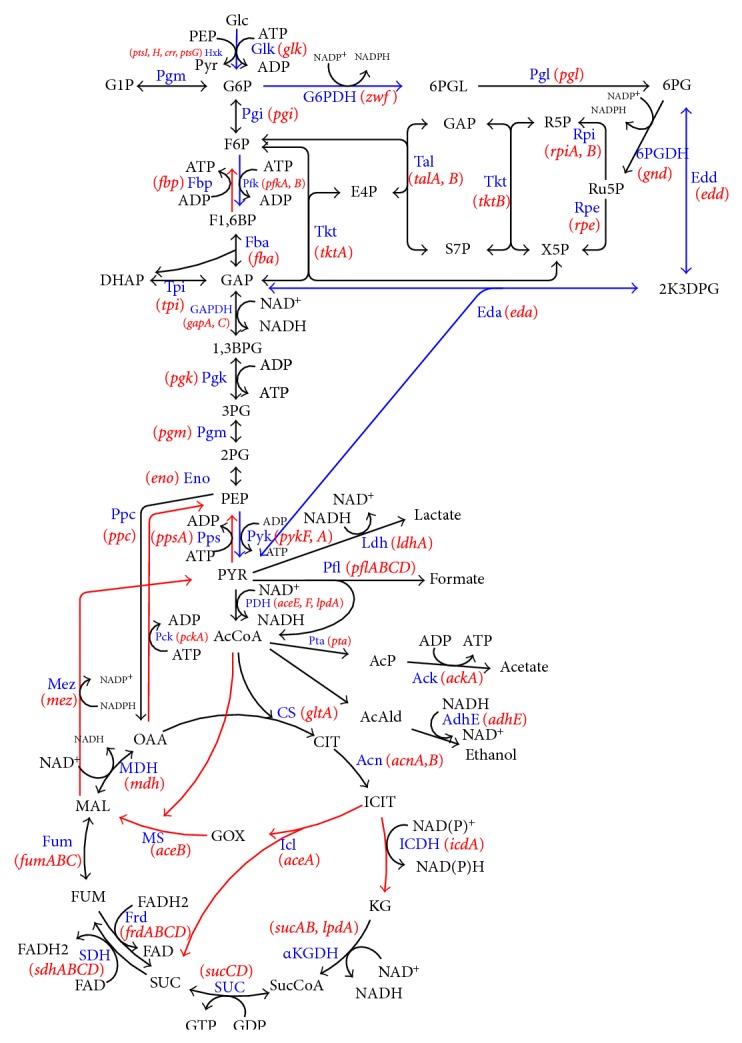
Pathway gene regulation of Cra.

 In addition to cAMP-Crp, which acts depending on the level of glucose concentration, the catabolite repressor/activator protein (Cra) originally characterized as the fructose repressor (FruR) plays an important role in the control of carbon flow in *E. coli *[164–166]. The carbon uptake and glycolysis genes such as *ptsHI*, *pfkA*, *pykF*, *zwf, *and *edd*-*eda *are reported to be repressed, while *ppsA*, *fbp*, *pckA*, *icdA*, *aceA, *and *aceB *are activated by the Cra protein [164, 166] (Figure 13). It has been shown that the genes such as *pfkA*, *pykF*, and *edd*-*eda *have Cra binding sites that overlap or follow the RNAP-binding site [167–170]. It is known that a mutant defective in the *cra *gene is unable to grow on gluconeogenic substrates such as pyruvate, acetate, and lactate [171]. This appears to be due to deficiency in the gluconeogenic enzymes such as Pps, Pck, some TCA cycle enzymes, the two glyoxylate-shunt enzymes, and certain electron transport carriers [171]. Molecular level research on *cra* gene expression has been made by several researchers using *lacZ*-transcriptional fusion [170, 172–175]. The gluconeogenic pathway is deactivated by the knockout of *cra *gene, and the carbon flow toward glycolysis and the glucose consumption rate are expected to increase since glycolysis pathway genes such as *ptsHI*, *pfkA, *and *pykF *are activated by the *cra *gene knockout. It has been shown that *cra *gene knockout enables the increase of the glucose consumption rate and thus improve the rate of metabolite production under certain culture conditions [176]. However, the regulation mechanism is complex, and it must be careful since *icdA, aceA, B*, and *cydB* genes are repressed, while *zwf *and *edd* gene expressions are activated, and thus ED pathway is activated by *cra* gene knockout [177]. Phue et al. [178] also studied the role of the *cra *gene in relation to high density cell cultures of *E. coliB *and *E. coliK*. 

### 4.7. Effect of Glucose Concentration on Gene Expressions in *E. coli*

**Figure 14:**
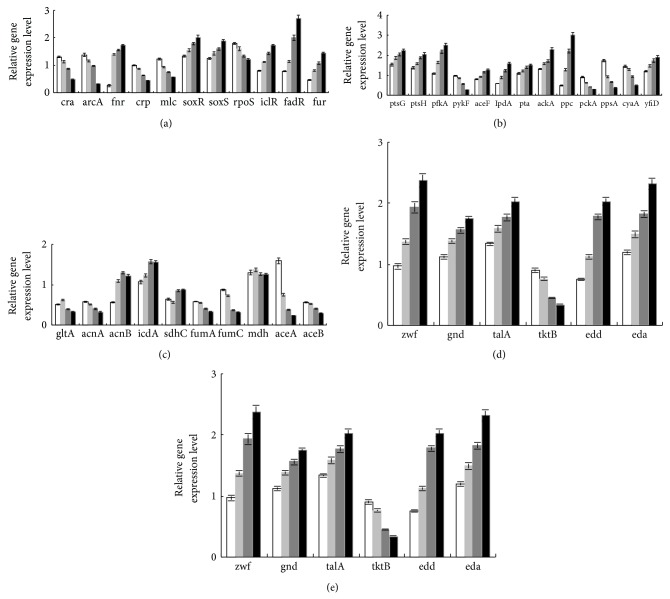
The effect of dilution rate on the gene transcript levels. (a) Global regulator genes; (b) PTS, glycolysis, anaplerotic pathway, *cyaA,* and *yfiD* genes; (c) TCA and glyoxylate pathway genes; (d) PP pathway genes; (e) Respiratory chain genes. White bar: 0.2; Light grey bar: 0.4; Dark grey bar: 0.6; Black bar: 0.7.

Let us consider how the culture condition such as glucose concentration affects the global regulators and metabolic pathway genes of wild type *E. coli *(BW25113) [179]. Table 1 shows the fermentation characteristics of the wild type *E. coli *for the continuous culture at different dilution rates, where it indicates that the specific glucose uptake rate, the specific acetate production rate, and the specific CO_2_ evolution rate (CER) increase as the dilution rate was increased [179].  Figure 14 shows the effect of the dilution rate (the specific growth rate) on gene transcript levels, where it indicates that in accordance with the increased specific glucose consumption rate, the transcript levels of *ptsG*, *ptsH*, and *pfkA* are increased as the dilution rate increased, where *cra* transcript level decreased and *crp* as well as *mlc* decreased accordingly. The decrease in* crp* is also coincident with the decrease in *cyaA* which encodes Cya. The transcript levels of *zwf*, *gnd*, *edd*, and *eda* increased as the dilution rate increased in accordance with the decrease in *cra*. The transcript level of *ppc* increased while *pckA* decreased as the dilution rate was increased. Moreover, the transcript levels of *fadR* and *iclR* increased, and *aceA* and *aceB* decreased as the dilution rate increased. In accordance with the increase in the specific acetate production rate, the transcript levels of *pta* and *ackA* increased. Further observation indicates that in accordance with the decrease in *rpoS* transcript level, *tktB*, *acnA*, and *fumC* decreased as the dilution rate increased, where this phenomenon will be discussed later for nutrient starvation.

## 5. Nitrogen Regulation

**Figure 15:**
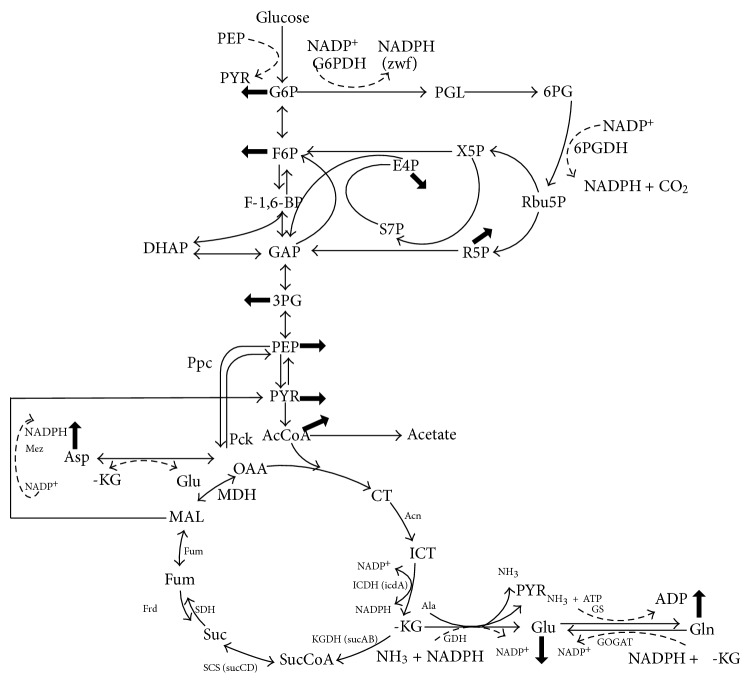
Central metabolic pathways and NH_3_-assimilation pathways.

**Figure 16:**
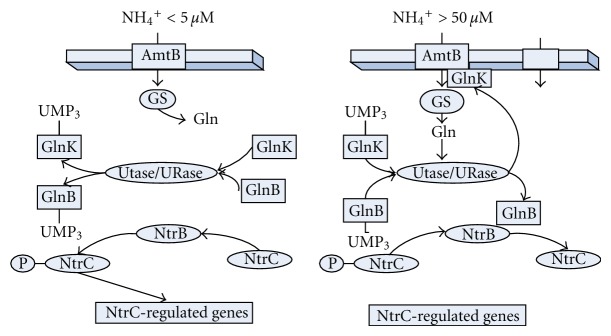
Ammonia assimilation under different NH_4_^+^ concentration.

Next to carbon (C) source metabolism, nitrogen (N) metabolism is also important in understanding the metabolic regulation. In *E. coli*, assimilation of N-source such as ammonia/ammonium (NH_4_^+^) using *α*-KG results in the synthesis of glutamate and glutamine (Figure 15). Glutamine synthetase (GS, encoded by *glnA*) catalyzes the only pathway for glutamine biosynthesis. Glutamate can be synthesized by two pathways through the combined actions of GS and glutamate synthase (GOGAT, encoded by *gltBD*) forming GS/GOGAT cycle, or by glutamate dehydrogenase (GDH encoded by *gdhA*) [180]. The GS/GOGAT cycle has a high-affinity for NH_4_^+^ (*K*_*m*_ < 0.2 mM for GS), and therefore it is dominant when nitrogen is scarce in the medium, whereas GDH has a low-affinity for NH_4_^+^ (*K*_*m*_ < 1 mM) and is utilized when sufficient nitrogen source is available in the medium. When extracellular NH_4_^+^ concentration is low around 5 *μ*M or less, ammonium enters into the cell via AmtB and is converted to glutamine by GS, and UTase uridylylates both GlnK and GlnB [181] (Figure 16). When extracellular NH_4_^+^ concentration is more than 50 *μ*M, the metabolic demand for glutamine pool rises, and UTase deuridylylates GlnK and GlnB. GlnK complexes with AmtB, thereby inhibiting the transport via AmtB, where GlnB interacts with NtrB and activates its phosphatase activity leading to dephosphorylation of NtrC and NtrC-dependent gene expression ceases [181] (Figure 16). The nitrogen intermediates such as glutamate and glutamine provide nitrogen for the synthesis of all the other N-containing components. About 88% of cellular nitrogen comes from glutamate, and the rest from glutamine [182]. The ATP required for the nitrogen assimilation using GS/GOGAT cycle under N-limiting condition accounts for 15% of the total requirement in *E. coli.* A significant amount of NADPH is also required for nitrogen assimilation [180, 182]. The other pathways involved in maintaining cellular nitrogen balance under specific conditions include aspartate-oxaloacetate and alanine-pyruvate shunts [183, 184].

It should be noted that carbon metabolism is not only controlled by carbon-derived signals, but also by the availability of nitrogen and other nutrient [185]. From the studies on interdependence of different metabolic routes, two of the major signal transduction systems of nitrogen and carbon metabolisms have been identified as P_II_, a small nitrogen regulatory protein and PTS as explained before. Because of the important roles in the regulatory functions, P_II_ and PTS can be regarded as the central processing units of N and C metabolisms, respectively. The P_II_ protein senses *α*KG and ATP, thus link the state of central carbon and energy metabolism for the control of N assimilation [185]. The glucose catabolism is modulated by the global regulators such as Cra, Crp, Cya, and Mlc while N assimilation is regulated by P_II_-Ntr system together with global regulators such as Crp, providing a novel regulatory network between C and N assimilation in *E. coli* [186]. The effects of C and N limitations on *E. coli* metabolism have been investigated for the continuous culture [187–191]. The C and N metabolisms may be linked by energy metabolism. It has been reported that the P_II_ protein controls N assimilation by acting as a sensor of adenylate energy charge, which is the measure of energy available for the metabolism. The signal transduction requires ATP binding to P_II_, which is synergistic with the binding of *α*KG. Moreover, *α*KG serves as a cellular signal of C and N status and strongly regulates P_II_ functions [192]. The studies on the C and N pathway interdependence have so far focused on the conversion of *α*KG to glutamate [193]. It is evident that the regulatory mechanism of this conversion is critical for the interdependence of C and N assimilation. 

**Figure 17:**
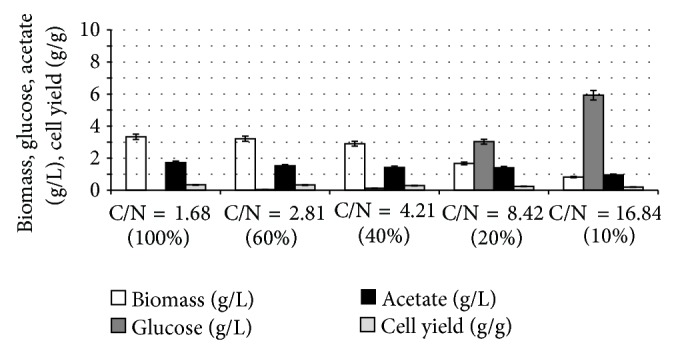
Effect of C/N ratio on the fermentation characteristics for the continuous culture at the dilution rate of 0.2 h^−1^.

Figure 17 shows the effect of C/N ratio on the fermentation characteristics during aerobic continuous culture at the dilution rate of 0.2 h^−1^, where the C/N ratio is the value of the feed substrate concentrations [193]. Figure 17 indicates that the glucose concentration increases, whereas the cell concentration decreases as C/N ratio increases. Figure 17 also shows that the glucose concentration is very low at 100% and 60% of N concentrations (C-limitation), whereas its concentration is high at 20% and 10% of N concentrations (N-limitation). Note that the specific glucose consumption rate as well as the specific acetate and CO_2_ production rates tended to increase as C/N ratio increases. 

**Figure 18:**
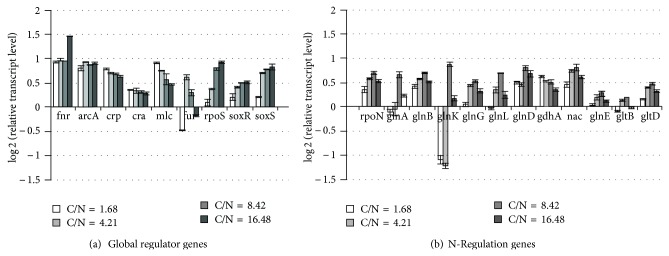
Comparison of the transcriptional mRNA levels of the wild-type *E. coli *genes cultivated at 100% (C/N = 1.68), 40% (C/N = 4.21), 20% (C/N = 8.42), and 10% (C/N = 16.8) N concentration.

In order to interpret the fermentation characteristics as shown in Figure 17, the relative mRNA levels are shown for different C/N ratios in Figure 18, where it shows that *crp* transcript level became lower as C/N ratio increases, which corresponds to the fact that cAMP-Crp level decreases as glucose concentration increases. In accordance with the change in *crp* transcript level, *mlc* level changed in a similar fashion [194].  Figure 18(a) also shows that the transcript levels of such genes as *soxR/S* and *rpoS *became higher as C/N ratio increases, which may be due to oxygen stress caused by higher respiratory activity for the former [188], along with nutrient stress for the latter [195]. 

The transcript level of *rpoN*, which encodes *σ* ^54^, increased as C/N ratio increases (Figure 18(b)). Figure 18(b) also shows that the expressions of *glnA, glnL, glnG,* and *gltD* genes changed in a similar fashion as *rpoN*, indicating the activation of GS-GOGAT pathway under N-limitation. The *glnB *gene which codes for P_II_ also changed in a similar fashion, while *glnD* which controls the uridylylation and deuridylylation shows somewhat different, but the trend seems to be similar (Figure 18(b)). P_II_ paralogue-encoding gene, *glnK* shows very high expressions at 20% and 10% of N-limitation (Figure 18(b)). The expression pattern of *nac* is similar to that of *rpoN*, whereas *gdhA* shows a reverse pattern, implying that *gdhA* is repressed by Nac (Figure 18(b)). The GDH pathway is favored when the organism is stressed for energy because GDH does not use ATP as does GS pathway [196]. Figure 18(b) shows the decreased expression of *gdhA *as C/N ratio increases. Liang and Houghton [197] investigated the effect of NH_4_Cl concentration on GDH and GS activities and showed the upregulations of GDH and transhydrogenase activities at lower NH_4_Cl concentration. 

**Figure 19:**
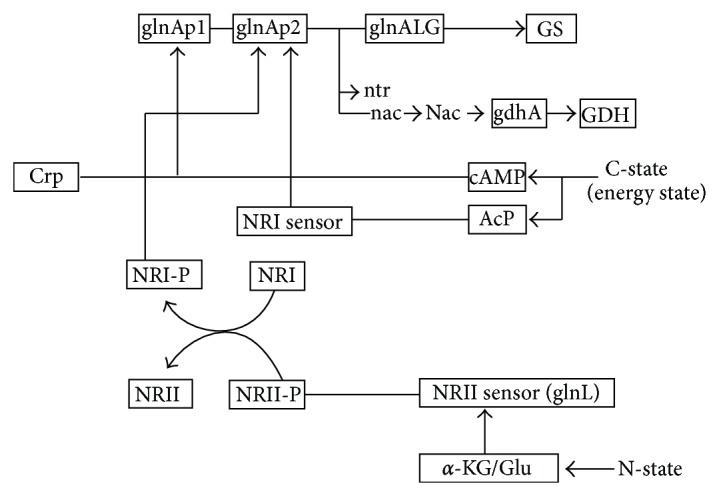
The interaction between nitrogen regulation and catabolite regulation.

The availability of nitrogen is sensed by P_II_ protein at the level of intracellular glutamine, where glutamine is synthesized by glutamine synthetase (GS) encoded by *glnA* and is transported mainly by GlnHPQ. The *glnHPQ* operon is under the control of tandem promoters such as *glnHp1* and *glnHp2*, where the former is *σ* ^70^ dependent, and the latter is *σ* ^54^ dependent and NtrC-P dependent [198, 199]. It has been shown that as the major transcriptional effector of the glucose effect, Crp affects nitrogen regulation [186]. Namely, *glnAp1* is activated by Crp with glutamine as N-source (Figure 19). Through *glnHPQ*-dependent signaling, Crp acts to decrease the amount of the phosphorylated NtrC activator, which in turn causes the decrease in *glnAp2* expression [186]. However, this regulation is more complex. It has been suggested that *σ* ^54^-dependent Ntr genes of *E. coli* form a gene cascade in response to N-limitation [200]. The central participants of Ntr response are NR_I_ (or NtrC) and NR_II_ (or NtrB), and RNA polymerase complexed to *σ* ^54^. NR_I_ is the transcriptional activator of *σ* ^54^-dependent promoters, while NR_II_ is a bifunctional protein that can either transfer phosphate to NR_I_ or control the dephosphorylation of NR_I_-phosphate. N-limitation results in the phosphorylation of NR_I_, which in turn stimulates the expression of *glnALG* operon. The expression of the *glnALG* operon is controlled by tandem promoters such as *glnAp1* and *glnAp2*, where *glnAp1* is a *σ* ^70^-dependent weak promoter, and its transcription can be activated by Crp and blocked by Ntr-P. On the other hand, *glnAp2* is transcribed by RNA polymerase (E*σ* ^54^) and is activated by Ntr-P. Therefore, *glnAp2* is responsible for activating *glnA* transcription under N-limitation [201]. 

It has been reported that there is no NR_I_-P binding sites in the *gdhA *regulatory region [202], and it is unlikely for NR_I_ to directly repress *gdhA* promoter [203]. As it has been shown that Nac is involved in the transcriptional repression of *gdhA* gene under N-limitation [203], Nac seems to repress *gdhA *gene as shown in Figure 18, where it shows that the transcript level of *gdhA* gene is lower, while *gltB* and *D* genes are higher under N-limitation as compared to C-limitation. NADPH is an important cofactor in GDH and (GS)-GOGAT activities and it has been reported that transhydrogenase plays some role in the regulation of these pathways [197]. Under N-limitation, the glutamate and glutamine synthetic pathways are expected to be repressed due to shortage of NH_3_ for those reactions, and thus NADPH is less utilized, resulting in overproduction of NADPH. Part of this may be converted to NADH by transhydrogenase, and the converted NADH together with other NADH formed may be utilized for ATP production through respiratory chain. 

*E. coli* possesses two closely related P_II_ paralogues such as GlnB and GlnK, where GlnB is produced constitutively, and it regulates the NtrB (NR_II_)/NtrC (NR_I_) two-component system [181]. It has been shown that the intracellular concentrations of NR_I_ and NR_II_ increase upon N-limitation [204–206]. The phosphorylated NtrC is an activator of various nitrogen-controlled genes such as *glnA *which codes for GS [200] and *glnK* encoding the second P_II_ paralogues [206]. The increased NR_I_, presumably in the phosphorylated form such as NR_I_-P activates the expression of *glnK* and *nac* promoters under N-limitation [207, 208]. Figure 18 shows that the transcript levels of *glnK* and *nac* genes increased as C/N ratio increases, where it has been reported that *glnK* and *nac* promoters are sharply activated when ammonia is used up [208]. The *gltBDF* operon which has been found to have binding affinity with global regulators such as Fnr and Crp in the promoter region [209].

**Figure 20:**
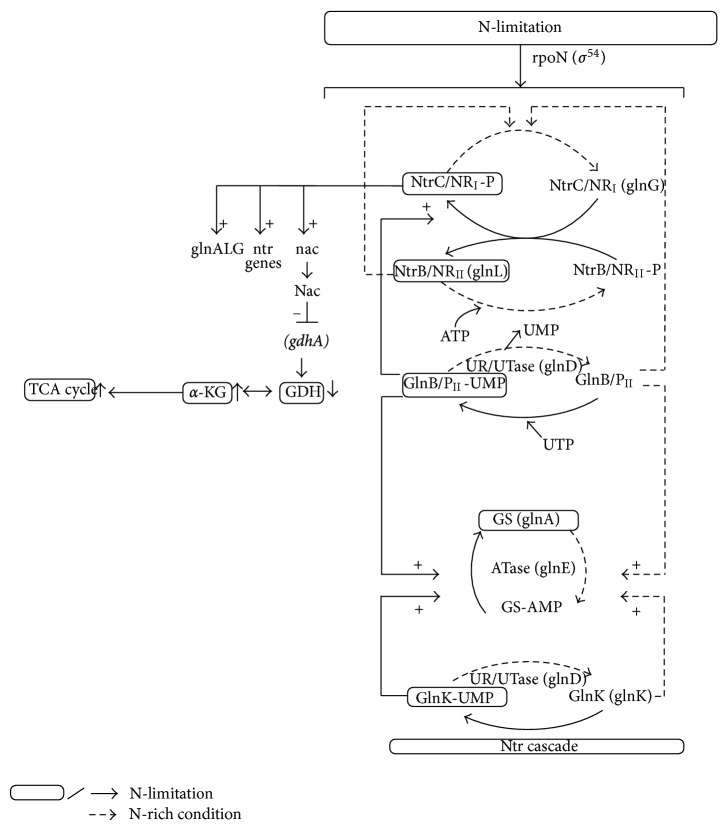
Overall mechanism of nitrogen assimilation in *E. coli* under C-limited (N-rich) and N-limited conditions.

The Ntr system is composed of four enzymes (Figure 20): a uridylyltransferase/uridylyl-removing enzyme (UTase/UR) encoded by *glnD *gene, a small trimeric protein, P_II_ encoded by *glnB*, and the two-component system composed of NtrB and NtrC. GlnD controls the activity of GS by adenylylation/deadenylation through a bifunctional enzyme adenylyltransferase (ATase), the *glnE* gene product [210–212]. The activity of GlnK becomes high under N-limitation and contributes to the regulation of NtrC-dependent genes [213]. It has been shown that on GS adenylation, ATase activity is regulated by UTase/UR and P_II_ such that upon nitrogen limitation, UTase covalently modifies P_II_ by addition of a UMP group at a specific residue and the resultant uridylylated form of P_II_ promotes deadenylylation of GS by ATase (Figure 20). Conversely, under N-rich condition, the uridylyl-removing activity of GlnD predominates, and the deuridylylated P_II_ promotes adenylation of GS by ATase. Adenylylation by ATase is promoted by deuridylylated P_II_ which is produced by UR action on P_II_ (UMP)_3_ under higher N-concentration (low C/N ratio) (Figure 20). These indicate that UTase/UR and P_II_ acting together sense the intracellular nitrogen status [214]. The P_II_ signal transduction proteins such as GlnB and GlnK are uridylylated/deuridylylated in response to intracellular glutamine level, where low intracellular glutamine level, signalling N-limitation, leads to uridylylation of GlnB [214]. GlnB is shown to be allosterically regulated by *α*-KG, and thus GlnB may play a role in integrating signals of C/N status. The NtrB/NtrC two-component system and GlnE which adenylylates/deadenylylates GS are the receptors of GlnB signal transduction [213]. It has been suggested that the carbon/cAMP effect is mediated through GlnB uridylylation [213]. 

The phosphorylated NR_I_/NtrC (NR_I_/NtrC-P) activates transcription from N-regulated *σ* ^54^-dependent promoters by binding to the enhancers [214–217]. P_II_ and the related GlnK protein control the phosphorylation state of NR_II_/NtrB by stimulating the phosphatase activity of NR_II_. The ability of GlnK and P_II_ to regulate the activities of NR_II_ is in turn regulated by the intracellular signals of C and N availability via allosteric control [215].

## 6. Phosphate Regulation

The phosphate (P) metabolism is also quite important from the energy generation and phosphorelay regulation points of view. The phosphorous compounds serve as major building blocks of many biomolecules and have important roles in signal transduction [65]. The phosphorus compounds serve as major building blocks of many biomolecules and have important roles in signal transduction [65]. The phosphate is contained in lipids, nucleic acids, proteins, and sugars and is involved in many biochemical reactions by the transfer of phosphoryl groups [218]. Moreover, phosphate metabolism is closely related to the diverse metabolisms such as energy and central carbon metabolisms [219]. All living cells sophisticatedly regulate the phosphate uptake, and survive even under phosphate-limiting condition [220, 221]. *Escherichia coli* contains about 15 mg of phosphate (P) per g (dry cell weight) [222]. Depending on the concentration of environmental phosphate, *E. coli *controls phosphate metabolism through Pho regulon, which forms a global regulatory circuit involved in a bacterial phosphate management [65, 223]. The PhoR-PhoB two-component system plays an important role in detecting and responding to the changes of the environmental phosphate concentration [224–226]. It has been known that PhoR is an inner-membrane histidine kinase sensor protein that appears to respond to variations in periplasmic orthophosphate (P_i_) concentration through interaction with a phosphate transport system, and that PhoB is a response regulator that acts as a DNA-binding protein to activate or inhibit specific gene transcription [65, 227–229]. The activation signal, a phosphate concentration below 4 *μ*M, is transmitted by a phosphorelay from PhoR to PhoB. Phospho-PhoB in turn controls Pho regulon gene expressions. PhoB is phosphorylated by PhoR under phosphate starvation or by PhoM (or CreC) in the absence of functional PhoR [230–236]. 

**Figure 21:**
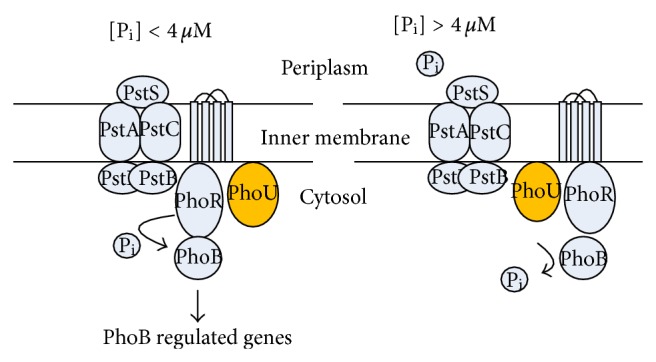
Molecular mechanism of phosphate regulation.

The *E. coli* Pho regulon includes 31 (or more) genes arranged in eight separate operons such as *eda, phnCDEFGHIJKLMNOP, phoA, phoBR, phoE, phoH, psiE, pstSCAB-phoU,* and *ugpBAECQ *[237]. When P_i_ is in excess, PhoR, Pst, and PhoU together turn off the Pho regulon by dephosphorylating PhoB. In addition, two P_i_-independent controls that may be formed of cross-regulation turn on the Pho regulon in the absence of PhoR. The sensor CreC, formerly called PhoM, phosphorylates PhoB in response to some (unknown) catabolite, while acetyl phosphate may directly phosphorylate PhoB [223]. When P_i_ is in excess, P_i_ is taken up by the low-affinity P_i_ transporter, Pit. Four proteins such as PstS, PstC, PstA, and PstB form an ABC transporter important for the high-affinity capture of periplasmic inorganic phosphate (P_i_) and its low-velocity transport into the cytosol [238]. These proteins are encoded together with PhoU as the *pstSCAB-phoU* operon. PstS is a periplasmic protein that binds P_i_ with high-affinity. PstC and PstA are inner membrane channel proteins for P_i_ entry, while PstB is an ATP-dependent permease that provides the energy necessary for P_i_ transport from periplasm to cytosol (Figure 21). When phosphate is in excess, the Pst system forms a repression complex with PhoR, and prevents activation of PhoB. PhoU and PstB are also required for dephosphorylation of phospho-PhoB under P-rich condition [239]. Indeed, PhoU is essential for the repression of the Pho regulon under high phosphate condition [64]. It may be considered that PhoU acts by binding to PhoR, PhoB or PhoR/PhoB, complex to promote dephosphorylation of phosphorylated PhoB or by inhibiting formation of the PhoR-PhoB complex [240]. 

It has been shown that* phoB *mutant does not synthesize alkaline phosphatase (*phoA* gene product) [241–246] and phosphate-binding protein (*pstS* gene product) [242, 245, 246]. It was observed that *phoU *expression changed depending on phosphate concentration of the *phoB* mutant [247]. Since the *phoA* gene mutation leads to the decreased content of membrane proteins or completely lacks them, mutations in the *phoB* gene result in the loss of alkaline phosphate and two membrane proteins [248]. Nesmeianova et al. [240] found that *phoB* mutation leads to the loss of polyphosphate kinase activity which catalyzes the synthesis of polyP in *E. coli*. Ault-Riché et al. [248] also found that the strains with deletion of *phoB *failed to accumulate polyP in response to osmotic stress or nitrogen limitation. Mutations in the *phoB* gene had no effect on *pepN *[249] and *lky* (*tolB*) expressions [250].

The expressions of the genes under the control of the PhoR-PhoB two-component system were found to be affected by the duration of P-limitation in response to phosphate starvation in *E. coli*. This means that the roles of the PhoR-PhoB two-component regulatory system seem to be more complex [226]. Since phosphate starvation is a relatively inexpensive means of gene induction in practice, the *phoA* promoter has been used for overexpression of heterologous genes [251]. A better understanding of the Pho regulon would allow for the optimization of such processes [238]. 

**Figure 22:**
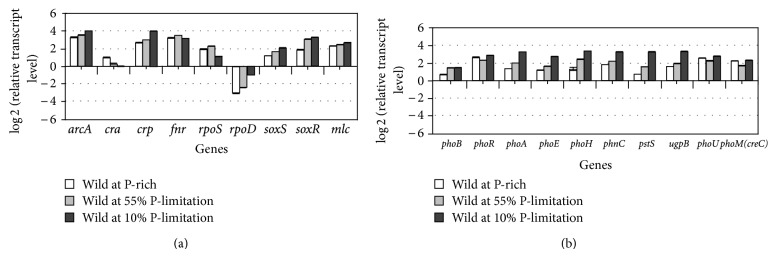
Comparison of the transcript levels of the wild-type *E. coli *cultivated with different P concentrations of the feed (100%, 55%, and 10%): (a) global regulatory genes and (b) PhoB regulatory genes.

Figure 22 shows the effect of P concentration on the transcript levels, where it indicates that *phoB* transcript level increased as P concentration decreases, and *phoB* regulated genes such as *phoA, phoE, phoH, phnC, pstS, *and* ugpB* were all increased in a similar fashion [252]. Note that *phoU* and *phoM* changed in a similar fashion as *phoR*, and also that the transcript level of *rpoD*, which encodes the RNA polymerase holoenzyme containing *σ* ^70^, increased in a similar fashion as PhoB regulatory genes [253]. 

**Figure 23:**
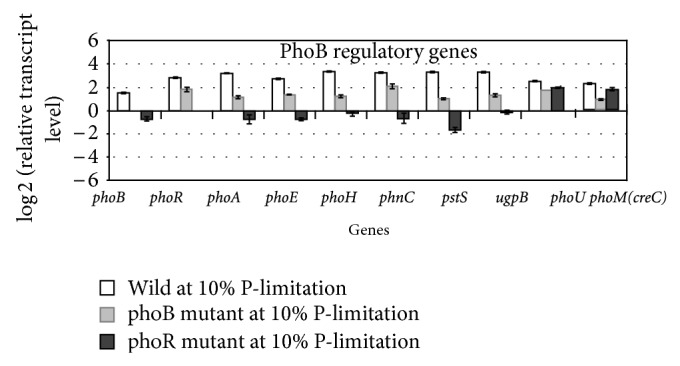
Comparison of the transcript levels of Pho regulon genes for the wild-type, *phoB,* and *phoR* mutants cultivated at 10% P-concentration.

The effect of *phoR* gene knockout on the selected gene transcript levels is also shown in Figure 23 where it indicates that the *phoB*-regulated genes such as *phoA, phoE, phoH, phnC, pstS,* and *ugpB *were more downregulated for the *phoR* mutant as compared to *phoB* mutant, whereas *phoU* and *phoM *(*creC*) were less affected by *phoR* gene knockout [252]. 

When cells enter into P_i_-starvation phase in the batch culture, the Pho regulon is activated and *σ* ^S^ starts to accumulate in the cytosol [65, 254, 255]. The promoters of the Pho genes are recognized by *σ* ^D^-associated RNA polymerase. A mutation in *rpoS*, significantly increases the level of AP (alkaline phosphatase) activity, and the overexpression of *σ* ^S^ inhibits it [256]. It has been reported that in *rpoS* mutant, the expression of AP was considerably higher than that in wild-type strain, implying that *σ* ^S^ is involved in the regulation of AP. Other Pho genes such as *phoE* and *ugpB* are likewise affected by *σ* ^S^. The *rpoS* may inhibit the transcriptions of *phoA, phoB, phoE,* and *ugpB*, but not that of *pstS* [256]. The *pst* may be transcribed by both *σ* ^S^ and *σ* ^D^. The Pho regulon is thus evolved to maintain a tradeoff between cell nutrition and cell survival during P_i_-starvation [256]. The previous reports suggest that the Pho regulon and the stress response are interrelated [255–260].

The presence of glucose or mutations in *cya *or cAMP receptor protein gene (*crp*) leads to the induction of *phoA* gene in *phoR* mutatnt. This induction requires the sensor PhoM (CreC) and the regulator PhoB [261]. However, PhoM (CreC) may not detect glucose per se, where it may detect an intermediate in the central metabolism. Therefore, *cya* or *crp* mutation may indirectly affect PhoM-(CreC-) dependent control. In addition to P_i_ control, two P_i_-independent controls may lead to the activation of PhoB. These two may be connected to control pathways in carbon and energy metabolisms, in which intracellular P_i_ is incorporated into ATP. One P_i_-independent control is the regulation by the synthesis of acetyl phosphate (AcP), where P_i_ is incorporated into ATP at Ack (acetate kinase) pathway. AcP may act indirectly on PhoB.

## 7. Oxygen Level Regulation

### 7.1. Effect of Oxygen Limitation on the Metabolism

 In addition to nutrient sources, oxygen level is also quite important from the metabolic regulation point of view. Global regulators such as Fnr and ArcAB are mainly responsible for the regulation of the availability of oxygen and other electron acceptors in the culture environment, where Fnr regulates the expressions of metabolic pathway genes under anaerobic condition [262], while ArcAB regulates under both anaerobic and microaerobic conditions [263, 264]. It has been shown that ArcA/B system exerts more significant effect on the cell metabolism under microaerobic condition than under aerobic or anaerobic condition. The effect of ArcAB system on the flux distribution at pyruvate node has been investigated based on the extracellular metabolite concentrations [265, 266]. It was shown that lactate can be overproduced by *arcA/fnr* double mutant [265] in a similar way as *pfl* gene knockout [267, 268]. 

Reoxidation of the reducing equivalents such as NADH generated by the oxidation of the energy source occurs in the respiratory chain under aerobic or microaerobic condition. In *E. coli*, NADH is oxidized in the respiratory chain via a coupled NADH dehydrogenase NDH-1 encoded by *nuo* or an uncoupled dehydrogenase NDH-2 encoded by *ndh*, and the electron flows into quinone and quinol pool. Quinol is then oxidized by either the cytochrome bo or the cytochrome bd terminal oxidase complex, which in turn passes the electrons to oxygen with concomitant production of water. The two terminal oxidases differ in their affinities for oxygen as well as in their H^+^/e-stoichiometries, where cytochrome bo oxidase has a low-affinity for oxygen and translocates two H^+^s per e^−^, while cytochrome bd has a high-affinity to oxygen and translocates one H^+^ per e^−^. The *cyoABCDE* operon is represses by both ArcA and Fnr, while *cydAB* operon is activated by ArcA and repressed by Fnr [269].

The microbial cells such as *E. coli* can generate energy as ATP under wide ranges of redox condition. The reducing equivalents such as NADH are reoxidized in the respiratory chain, where oxygen, nitrate, fumarate, and dimethyl sulfoxide, and so forth, are the electron acceptors. This process is coupled to the formation of a proton motive force (PMF), which is utilized for ATP generation from ADP and P_i_. In the absence of oxygen, or other electron accepters, ATP is generated via substrate level phosphorylation through the process of degradation of carbon source in the metabolic pathways. Under such fermentation condition, the cell such as *E. coli *excretes such metabolites as lactate, ethanol, succinate, and formate (CO_2_ and H_2_ as well) as well as acetate, where the relative production rates for these metabolites are governed by the demand for redox neutrality. The succinate is formed from PEP via Ppc. Pyruvate serves as a common substrates for pyruvate formate-lyase (Pfl) and the pyruvate dehydrogenase complex (PDHc), and this branch point involves the cleavage of PYR. The expressions of* pfl* genes which encode Pfl is activated by ArcA and Fnr, and it becomes higher at lower oxygen concentrations, whereas *aceE,F* which encode *α* and *β* subunits of PDHc is repressed by ArcA under oxygen limited condition. At the branch point of AcCoA, the product of both Pfl and PDHc reactions, is converted to either acetate and ethanol or subsequently undergo further oxidation in the TCA cycle. The interconversion of Pfl between inactive and active glycyl radical-bearing species occurs at low oxygen concentration and is controlled by the activities of the iron-sulfur protein, Pfl activase, and the product of the *adhE* gene, Pfl deactivase [270, 271]. The active glycyl radical form of Pfl is irreversibly destroyed by molecular oxygen and hence must be either protected from oxygen damage or converted to the inactive, oxygen-stable species during the transition between anaerobiosis and aerobiosis [272, 273].

### 7.2. Regulation by ArcA/B System

The Arc (anoxic respiration control) system, composed of ArcA, the cytosolic response regulator, and ArcB, the membrane bound sensor kinase, regulates the TCA cycle genes depending on the oxygen level or redox state. The ArcB protein has three cytoplasmic domains: a primary transmitter domain (H1) containing a conserved His292, a receiver domain (D1) containing a conserved Asp576, and a secondary transmitter domain (H2) containing a conserved His717 [272, 274–276]. The primary transmitter domain of ArcB is autophosphorylated at His292 at the expense of ATP [277, 278]. The phosphoryl group is then sequentially transferred to Asp576 and His717 and from there to Asp54 of ArcA. However, the phosphoryl group on His292 could also be directly transferred to ArcA at a very low rate [277]. On the other hand, the phosphoryl group from His717 could also be transferred to ArcA, but this transfer is regulated by redox conditions [279]. Namely, upon stimulation by the redox state, ArcB undergoes autophosphorylation, and the phosphoryl group is transferred to ArcA by the His→Asp→His→Asp phosphorelay [280]. Consequently, the phosphorylated ArcA binds to the promoter regions of the TCA cycle and other genes. It has been reported that ArcA, when phosphorylated, represses the expressions of the genes involved in the TCA cycle and the glyoxylate shunt genes such as *glt*A, *acn*AB, *icd*A, *suc*ABCD, *sdh*CDAB, *fum*A, *mdh*, and *aceA, B* [281–286]. Moreover, the genes which encode the primary dehydrogenases such as *glpD*, *lctPRD, aceE,F* and *lpdA* are also repressed by ArcA [287–289]. *Escherichia coli* possesses two terminal quinol oxidases in the respiratory chain. The genes *cyo*ABCDE, which encode cytochrome o oxidase that has a low oxygen affinity and mainly functions under aerobic condition, are repressed by ArcA [290]. On the other hand, the *cyd*AB genes which encode cytochrome d oxidase that has high oxygen affinity are activated by ArcA [287, 291, 292].

Alexeeva et al. [263] investigated the effects of different oxygen supply rates on the catabolism in *arc*A mutant. It was shown that ArcAB system exerts more significant effect on the cell's catabolism under microaerobic condition than under aerobic or anaerobic condition. A strong link is demonstrated between redox ratio (NADH/NAD^+^) and acetate overflow in *E. coli* [293]. It was shown that the commencement of acetate overflow occurred above the critical NADH/NAD^+^ ratio of 0.06 [293]. Moreover, acetate overflow is delayed by the expression of heterologous NADH oxidase (NOX), an enzyme that serves to reduce the NADH/NAD^+^ ratio [293]. The redox state has been reported to trigger the Arc regulon [294, 295].

**Figure 24:**
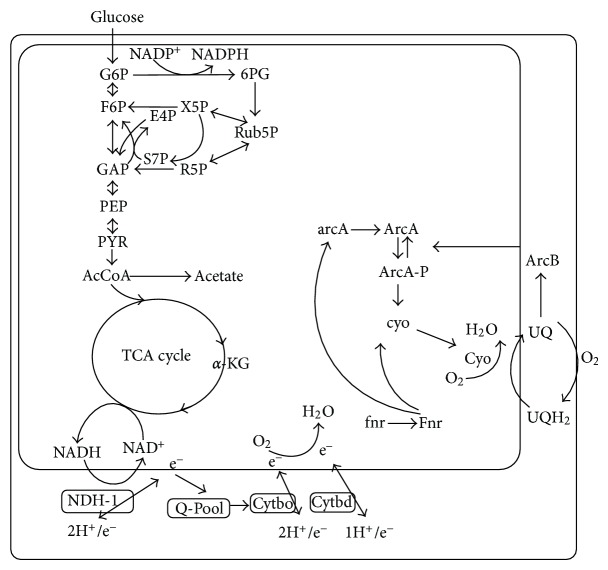
Fnr and ArcA/B and respiratory chain regulation.

Since phosphorylated ArcA represses TCA cycle genes, the *arcA* gene deletion activates the TCA cycle, resulting in the reduction in the acetate formation [293]. The NADH oxidation by the expression of NOX in the *arc*A gene knockout mutant further reduced the acetate formation, resulting in the increased recombinant protein production [293]. Since TCA cycle is the main source of energy generation and provides important precursors for amino acids such as glutamate, and lysine, it is of practical interest to enhance the TCA cycle activity. As stated above, the *arc*A/B genes knockout in *E. coli* transcriptionally activates the TCA cycle and overproduces NADH, which may in turn repress the TCA cycle by its allosteric regulation. Moreover, it has been reported that ArcAB does not control the TCA cycle under aerobic condition due to the fact that oxidized quinone electron carriers inhibit autophosphorylation of ArcB, and it can not transphosphorylate ArcA [294] (Figure 24). As expected from the above mentioned regulation, the TCA cycle is activated by *arcA/B* gene knockout, which then causes higher NADH/NAD ratio, which in turn represses TCA cycle activity [296]. Vemuri et al. [296, 297] considered to express heterologous *nox* gene to oxidize NADH, and in turn activate TCA cycle, while nicotinic acid and Na nitrate may also activate TCA cycle [298]. 

Since the TCA cycle is the source of energy generation and provide some of the precursors for the cell synthesis, the activation of the TCA cycle may lead to the improvement of ATP production for the cell growth and/or the TCA cycle-related metabolite productions in practice. It should be noted that the activation of TCA cycle reduced the acetate production rate, which is the common obstacle for the metabolite production using *E. coli*. It should, however, also be noted that the activation of the TCA cycle caused the decrease in the cell yield due to higher production of CO_2_ in the TCA cycle. This may be overcome by activating the glyoxylate pathway by *fadR* gene knockout [299], and so forth. It is controversial whether the cell metabolism is controlled to maximize ATP generation or cell synthesis, and so forth [300].

### 7.3. Fnr and Nar Systems

Respiration is a fundamental cellular process utilizing different terminal electron acceptors such as oxygen and nitrate. The ability to sense these electron acceptors is a key for the cells to survive. *Escherichia coli* is a metabolically versatile chemoheterotroph grown on a variety of substrate under various oxygen concentrations with fumarate or nitrate, replacing oxygen as terminal electron acceptor under anaerobic condition [301]. Many bacteria utilize oxygen as the terminal electron acceptor, but they can switch to other acceptors such as nitrate under oxygen limitation. In *E. coli*, this switch from aerobic to anaerobic respiration is controlled by Fnr (fumarate and nitrate reduction), where it was identified by Spiro and Guest [302]. Under oxygen limitation, Fnr binds a [4Fe-4S]^2+^ cluster and becomes a transcriptionally active dimeric form.

*Escherichia coli* possesses sensing/regulation systems for the rapid response to the availability of oxygen, redox state as represented by NADH/NAD^+^ ratio, and the presence of other electron acceptors. Those regulation systems channel electrons from donor to terminal acceptors. The pyridine nucleotides such as NADH and NAD^+^ function as the important redox carriers involved in the metabolism. These coenzymes not only serve as electron acceptors in the breakdown of substrates, but also provide the reducing power for the redox reactions in the anaerobic and aerobic respirations. A balance for oxidation and reduction of these nucleotides is regulated for catabolism and anabolism, since the turnover of the nucleotides is very high compared to their concentrations [303]. Under anaerobic condition, the reoxidation of NADH and the formation of reduced compounds occur, whereas NADH oxidation is coupled to the respiration by electron transfer under aerobic or nitrate respiration. 

The metabolic regulation is made by the binding of dimeric Fnr to the promoter regions of the relevant genes with affinities depending on the redox state [304]. The ability of Fnr to bind DNA is regulated by the change in equilibrium between monomeric apo Fnr (inactive) and dimeric Fnr (active)* in vivo*. The active form of Fnr binds to DNA to regulate the corresponding genes under anaerobic condition. Molecular oxygen can oxidize the ion-sulfer cluster of the corresponding region, resulting in monomerization of the protein and subsequent loss of its ability to bind DNA [305]. Nar (nitrate reduction) plays a role when nitrate is present, and belongs to the two-component redox regulation systems, where it comprises a membrane sensor (NarX) that may act as a kinase causing phosphorylation of the regulator (NarL) under certain conditions. The Nar system activates such genes as nitrate reduction encoding nitrate and nitrite reductases and represses such genes as fumarate reductase genes. 

The sequence of the *fnr* gene revealed that it encodes a protein which shows a significant homology to CAP/Crp (for catabolic activator protein). However, a number of significant differences between the two proteins have been investigated. Fnr is a monomeric protein, and it does not have the conserved group of surface residues that interact with cyclic AMP. It contains an oxygen labile iron-sulfur center as a sensor element for anaerobiosis [282, 306–308]. Several studies have been conducted on the structure and gene sequence for Fnr and Crp proteins. From these studies, it was found that both Fnr and Crp proteins possess almost similar structure and gene sequence. The genes that are controlled by these two global regulators have similar binding sites [308–310]. Even if some mutation changed the structure of proteins, the mutation in Fnr protein could convert to Crp protein, and similarly Crp protein could convert to Fnr protein [302]. It may be also considered that both Crp and Fnr proteins can form heterodimer, which might not allow both of them to function properly [306–308]. Then, the absence of Fnr protein or gene allows Crp protein to bind more effectively to the target gene sequence. 

Since Fnr is known to activate *frd* and *pfl* genes, the *fnr* mutant produced less succinate and formate [311]. Although *arcA* is known to be activated by Fnr, the regulation mechanism is somewhat complicated (Figure 24). Namely, *cyo* and *cyd *genes are repressed, by Fnr, while *cyo* is repressed, and *cyd* is activated by ArcA. The *fnr* mutant shows a decreased gene expression of *arcA*, and an increased gene expressions of both *cyoA *and *cydB*. This implies that the activated cytochrome oxydase increased quinone pool, which inhibits ArcB phosphorylation and in turn decreases phosphorylation of ArcA, where *arcA* gene expression also decreases due to *fnr* gene knockout. 

### 7.4. Effect of Excess Oxygen on the Metabolism

On the other hand, the microbial cell responds to oxidative stress by inducing antioxidant proteins such as superoxide dismutase and catalase [312]. The well characterized pleiotropic regulators of the antioxidant responses are the OxyR and SoxR [313]. The activation of both proteins results in the transcriptional enhancement of sets of genes whose products relieve the stress by eliminating oxidants and preventing or repairing oxidative damage [313]. SoxR is a member of the MerR family of metal-binding transcription factors, and it exists in solution as a homodimer with each subunit containing a [2Fe-2S] cluster. These clusters are in the reduced state in inactivated SoxR, and their oxidation activates SoxR as a powerful transcription factor [314]. The active form of SoxR activates transcription of *sox* gene. The *sox* gene product, SoxS, belongs to the AraC/XylS family of DNA-binding transcription factors [315]. SoxS regulates the expressions of more than 17 genes or operons [316–320]. 

Oxygen derivatives such as superoxide (O_2_^•−^), hydrogen peroxide (H_2_O_2_), and the hydroxyl radical (^•^OH) are usually generated as toxic by-products of aerobic metabolism in a cascade of monovalent reductions from molecular oxygen. Although these are not so reactive *per se*, O^•−^ and H_2_O_2_ have been reported to cause severe cell damage. H_2_O_2_ along with Fe^2+^ via the Fenton reaction produces ^•^OH, which can react with any macromolecule such as protein, membrane constituents, and DNA [321, 322]. O_2_^•−^ exacerbates the Fenton reaction by increasing the intracellular pool of “free iron,” for instance, by releasing iron from O_2_^•−^-oxidized [4Fe-4S] clusters. O_2_^•−^ may also react with nitric oxide (^•^NO), producing highly reactive peroxynitrite (ONOO^−^), which can generate ^•^OH. Despite their potential toxicity, reactive oxygen species (ROS) at low concentration have been shown to be actively involved in the cell's life and, therefore, should not be entirely eliminated. Potent basic defense systems maintain ROS at harmless levels but cannot deal with sudden increases in ROS production. This creates an imbalance between production and elimination, referred to as oxidative stress. 

Early studies using two-dimensional gel electrophoresis to analyze variations in protein expressions have shown that the synthesis of more than 80 proteins was activated in response to oxidative stress [312]. Some of these induced proteins are identified as possessing fundamental antioxidant functions, for example, superoxide dismutase and catalase. The search for mutants with altered antioxidant defenses led to the isolation and characterization of pleiotropic regulators that operate as redox-regulated genetic switches [315, 323, 324]. The best characterized pleiotropic regulators of the antioxidant responses are the OxyR and SoxR proteins [313]. Both proteins have the remarkable ability of directly transducing oxidative signals to genetic regulation. Both proteins are expressed constitutively in an inactive state and are transiently activated in cells under specific types of oxidative stress. The activation of the OxyR and SoxR proteins results in the transcriptional enhancement of sets of genes (regulons) whose products relieve the stress by eliminating oxidants and preventing or repairing oxidative damage [313]. 

The two enzymes involved in the oxidative PP pathway, G6PDH and 6PGDH that provide NADPH for biosynthesis, are significantly affected in both *soxR* and *soxS* mutants [325]. The activities of G6PDH and 6PGDH decreased in both *soxR* and *soxS* mutants, compared to the parent stain. The downregulations of these two enzymes agreed with the slower growth rates in both mutants, since these enzymes are known to be under growth rate-dependent regulation [326]. The downregulation of *zwf* gene in both mutants is also due to the effects of *soxS* and *soxR* genes deletion, since *zwf* is a member of *soxRS* and multiple antibiotic resistance (*mar*) regulons. Thus, unlike *gnd*, *zwf* expression is transcriptionally activated by SoxS during episodes of oxidative stress [323, 327]. The *pntA* (membrane bound transhydrogenase) transcripts which is involved in NADPH generation [328], are upregulated in both *soxR* and *soxS* mutants. This may be due to the down-regulation of NADPH-generating enzymes such as G6PDH and 6PGDH in PP pathway, and ICDH in TCA cycle, since NADPH plays a significant role to reduce oxidative stress [323]. 

## 8. Acid Schock or the Effect of pH

The acid barrier of the stomach represents a strong challenge for many pathogenic enterobacteria. Enteric bacteria that cause disease in the human intestine endure a transient but extreme acid condition in the stomach. The normal stomach shows an acid environment at around pH 2 with an emptying time of about 2 h [329]. Unlike acid sensitive *Vibrio cholerae*, *Escherichia coli, *and *Shigella* have a potent acid resistant systems that are able to withstand at low pH at around 2 for at least 2 h [330, 331]. *E. coli* possesses a level of acid resistance rivaling that of gastric pathogen *Helicobacter Pylori* [332]. As such, it is quite important to understand the cell metabolism in relation to acidic condition from both medical and fermentation points of view. The molecular and physiological response to acid stress has been thus the subject of intense investigation (Table 3) [333, 334]. 

**Figure 25:**
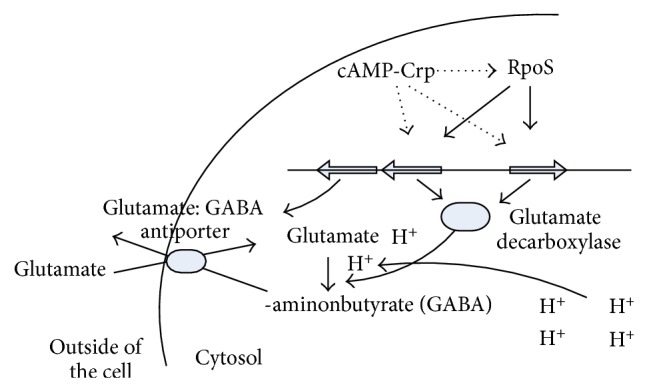
The role of glutamate decarboxylase for acid resistance.

Several acid stress response systems that can protect *E. coli *from acidic condition have been investigated [333–336]. Some of these depend on the available extracellular amino acids such as glutamate, arginine, and lysine, where the intracellular proton is consumed by the reductive decarboxylation of the amino acid followed by the excretion of the product such as *γ*-amino butyric acid (GABA) from cytoplasm to the periplasm by a dedicated antiporter that also imports the original amino acid [333] (Figure 25). *E. coli* cells have been demonstrated to exhibit acid resistance by such genes as *gadAB* which encodes glutamate decarboxylase and *gadC* which encodes glutamate/GABA antiporter. Glutamate decarboxylase production has been shown to increase in response to acid, osmotic, and stationary phase signals. The *gadA* and *gadB* genes for glutamate decarboxylase isozymes form a glutamate-dependent acid response system, where the process of decarboxylation consumes an intracellular proton and helps maintain pH homeostasis. It has also been known that there exists similar acid resistant systems for the case of using arginine instead of glutamate by arginine decarboxylase, where the antiporter is AdiC in this case [330, 331, 337, 338], and for the case of using lysine by lysine decarboxylase [338]. Note that the cells grown in media rich with amino acids such as LB are acid resistant [333].

In the typical batch culture, organic acids are most accumulated at the late growth phase or the stationary phase, and it has been known that GadA and GadB proteins increase in response to stationary phase and low pH [339]. The sigma factor *σ* ^S^ or RpoS which increases its amount at the late growth phase and the stationary phase as well as Crp are involved in acid resistance [330, 333]. As would be implied by the involvement of Crp, the resistance system is repressed when glucose is present. Moreover, it has been known that FoF_1_ proton-translocating ATPase is involved in this system [336]. The FoF_1_ ATPase is utilized for the protons in the periplasm move into the cytosol across the cell membrane producing ATP from ADP and P_i_ by the negative proton motive force (PMF) [340]. Since the basic problem of acid stress is the accumulated proton in the cytosol, this proton can be pumped out through FoF_1_ ATPase by hydrolyzing ATP and reversed proton move due to positive PMF at low pH such as pH 2 or 3 [336]. Without amino acid available in the media, this acid response system is activated by utilizing FoF_1_ ATPase [335, 341], where the positive proton motive force (PMF) pumps extra protons (H^+^) from the cytoplasm with consumption of ATP [336]. Namely, PMF is operated in the reverse direction as compared to the case of producing ATP. 

Table 2 shows 11 regulators involved in regulating glutamate-dependent acid resistance. In the typical batch culture, the *gadA/BC* loci can be induced during growth in acidic minimal media (pH 5.5) or in the stationary phase regardless of pH [333]. However, in a complex media such as LB, locus is not induced until the culture enter into stationary phase.

**Figure 26:**
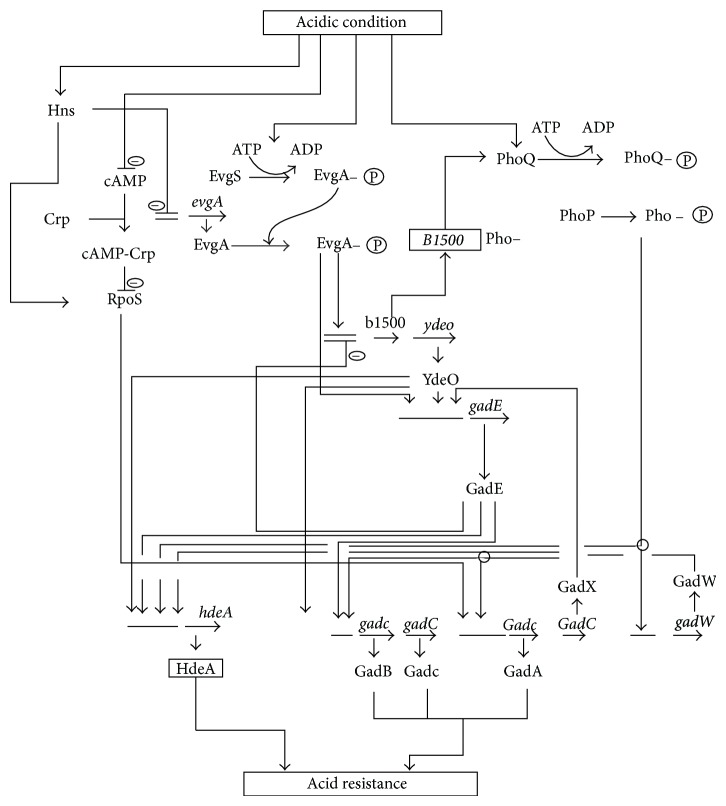
Acid resistance mechanism under acidic condition.

The expressions of *gadA/BC* genes are under control of GadE and the response regulator RcsB [342], where RcsB is part of the RcsCDB phosphorelay, a signal transduction system conserved in the members of *Enterobacteriaceae*. The RcsB can be also activated independently of the phosphorelay, by binding of different coregulators such as RcsA (main one), RmpA, TviA, and PhoP [342]. As shown in Figure 26 [343], EvgS is a sensor kinase and phosphorylates EvgA, where the phosphorylated EvgA activates *gadE *gene as well as *ydeO*, where YdeO also regulates *gadE* gene. It has also been shown that small membrane protein B1500 connects the signal transduction cascade between EvgS/EvgA and PhoQ/PhoP, where *b1500* is located upstream of *ydeO *and under control of EvgA [344]. It has been known that Mg^2+^ turns off the PhoQ/PhoP system [345]. Moreover, the phosphorylated PhoP activates *gadW*, where GadW activates *hdeA* and *gadA*, where *hdeA* is under the control of GadW, PhoP-P, and GadW. EvgA regulates at least eight genes related to acid resistance such as *ydeP, b1500,* and *ydeO* [346].

It has been shown that an acid pH lowers cAMP levels in exponentially growing cells in the minimal glucose medium. This may elevate RpoS that would drive increased expression of *gadX*. However, GadW represses RpoS synthesis at acidic condition, and in turn GadX synthesis. GadX, when not repressed by GadW, is acid induced due to changes in cAMP. GadW is also acid induced when it is not repressed by GadX. GadX directly binds to the *gadW* promoter region. GadX and GadW collaborate to repress *gadA* and *gadBC* expressions under alkaline conditions [347]. The GadX-GadW regulon has also been investigated by DNA microarray [348].

As explained before, the two-component system of EnvZ (sensor) and OmpR (regulator) regulates porin expression, where OmpR may be a key regulator for acid adaptation, and thus *opmR* mutant is sensitive to acid exposure [334]. It has been known that the level of OmpC increases with the increased osmolarity when cells are growing in neutral or alkaline media, whereas the level of OmpF decreases at high osmolarity [349]. It has also been known that these porin proteins play important roles at acidic condition.

The acid-inducible *asr* gene is reported to be regulated by the two-component systems PhoR/B which controls *pho* regulon in response to phosphate starvation, and thus PhoB-PhoR deletion mutant fails to induce *asr *gene expression [350]. It has also been suggested that H^+^ might activate a sensor protein PhoR in the periplasm [350] directly or via its acceptor.

Under anaerobic condition, additional genes like *ackA, lpdA*, and so forth, as well as *hdeA* and *ompT*, and so forth, are induced. In order to avoid deleterious concentration in the cell caused by the production at low pH, *ldhA* is induced by acid in order to produce lactate instead of more harmful acetate plus formate [351].

## 9. Heat Shock Response

The organisms respond to a sudden temperature upshift by increasing the synthesis of a set of proteins. This phenomenon is called the heat shock response. The research on heat shock response of a microorganism contributes to the variety of practical applications such as temperature-induced heterologous protein production [352, 353] and simultaneous saccharification and fermentation (SSF) [354]. The heat shock proteins play roles in the assembly and disassembly of macromolecular complex such as GroE [355], the intracellular transport such as Hsp70 [356], transcription such as *σ* ^70^ [357], proteolysis such as Lon [358], and translation such as lysyl-tRNA synthetase [359]. The heat shock response in *E. coli *is mediated by E*σ* ^32^ [360], and it has been known that at least 26 genes are induced by heat shock [361], where E denotes the RNA polymerase holoenzyme. Among them, *groEL, dnaK*, and *htpG* are the genes which code for the major chaperones such as Hsp 60, Hsp 70, and Hsp 90. The ClpP, Lon, and HtrC are involved in the proteolysis. DnaK, DnaJ, GrpE, and RpoH are involved in the autoregulation of heat shock response [362–365]. It has been known that DnaK prevents the formation of inclusion bodies by reducing aggregation and promotion of proteolysis of misfolded proteins [366]. A bichaperone system involving DnaK and ClpB mediates the solubilization or disaggregation of proteins [367]. GroEL operates protein transit between soluble and insoluble protein fractions and participates positively in disaggregation and inclusion body formation. Small heat shock proteins such as IbpA and IbpB protect heat-denatured proteins from irreversible aggregation and have been found to be associated with inclusion bodies [368, 369].

It has been reported on the molecular mechanisms of heat shock proteins [370, 371]. Hoffmann et al. [353] investigated the metabolic adaptation of *E. coli *during temperature-induced recombinant protein production and showed that cAMP/Crp-controlled LpdA of the pyruvate dehydrogenase complex (PDHc) and SdhA in the TCA cycle are induced four times, reaching a maximum at 1 h after temperature upshift. It is also shown that the TCA cycle enzymes such as ICDH and MDH are initially less produced but regained to their respective preshift values about 30 min after the temperature upshift. Gadgil et al. [372] investigated the effect of temperature downshift from 37°C to 33 and 28°C on gene expressions in *E. coli*. This kind of investigation is useful in analyzing the metabolic changes and investigating the effects of gene modification for strain improvement [373]. 

Upon temperature upshift from 37°C to 42°C, the expression of *rpoH* is upregulated, and the expressions of *dnaK, groL, groS, htpG,* and *ibpB* were upregulated [374], which are known to be under control of sigma factor (*σ* ^32^). It was also shown that *arcA* gene expression was upregulated, and that the expression of *crp* gene was upregulated, and the expression of *lpdA*, which is under control of Crp, was also upregulated [353, 374]. 

To survive, cells have to control gene expressions precisely in response to the changes in the growth environment. The microorganism such as *E. coli* attains this primarily at the transcription level. To control the initiation of the specific transcription, the cell uses diverse mechanisms including various sigma factors. The classical heat shock regulon has been shown to be under the control of *σ* ^32^ transcription factor, the product of *rpoH* gene [375]. The regulation of the sigma factor (*σ* ^32^) is complex and depends on the feedback control loops involving the *dnaK *chaperone and temperature-induced changes in mRNA secondary structure [376]. The relative levels of the major heat shock genes such as *dnaK*, *groS*, *groL*, *ibpB*, *lonA*, and *htpG* were upregulated after the temperature upshift. The expressions of heat shock genes such as *dnaK, groL, *and* ibpB* increased in the early induction phase (first 10–20 minutes) and then declined. In* E. coli*, heat shock protein synthesis rates peak at about 5~10 min after the temperature upshift and then declined to a new steady-state levels [377]. The heat shock response is made transcriptionally, where it has been known that the RNA polymerase core (E) binds to new initiation subunit *σ* ^32^ [378], and the resulting holoenzyme E*σ* ^32^ transcribes only heat shock genes [379], which have promoter sequences that differ from those transcribed by E plus *σ* ^70^, the normal vegetative initiation factor [380]. The transcription factor *σ* ^70^ is itself a heat shock protein, and the increase in its concentration after heat shock may contribute to its decline in heat shock protein synthesis. Moreover, other heat shock proteins, in particular the *dnaK* gene product contributes to the shutoff, since the mutations in their genes prolong the high level synthesis of heat shock proteins [381]. The heat shock response must be tightly regulated in order to allow rapid changes in heat shock protein synthesis rates. Although the level of mRNA transcribed from the *rpoH* gene increases after heat shock, their increases may be insufficient and too slow to be the sole explanation of the rapid effect of the heat shock. It has been shown that the concentration of active *σ* ^32^ limits the expression of heat shock genes, and that the stability of *σ* ^32^ is modulated [377]. 

Because of the rapid turnover (half-life of less than 1 min), the cellular concentration of *σ* ^32^ is very low at normal temperature and is limited for the transcription of the heat shock gene. Upon temperature upshift, *σ* ^32^ becomes transiently stabilized until the beginning of the shutoff phase of the heat shock response. The heat shock response is induced as a consequence of declining *σ* ^32^ levels and inhibition of *σ* ^32^ activity. Stress-dependent changes in heat shock gene are mediated by the antagonistic action of *σ* ^32^ and negative modulators which act upon *σ* ^32^. These modulators are the DnaK chaperone system which inactivates *σ* ^32^ by direct association and mediates its degradation by proteases [382]. Degradation of *σ* ^32^ is mediated mainly by FtsH and ATP dependent metalloprotease within the inner membrane. The heat shock proteins increased immediately after the temperature upshift, reached a maximum 5–15 min later and decreased to preshift values largely within 1 h, while the maximum induction of many heat-shock proteins including DnaK and HtpG reached at least 30 min later. 

**Figure 27:**
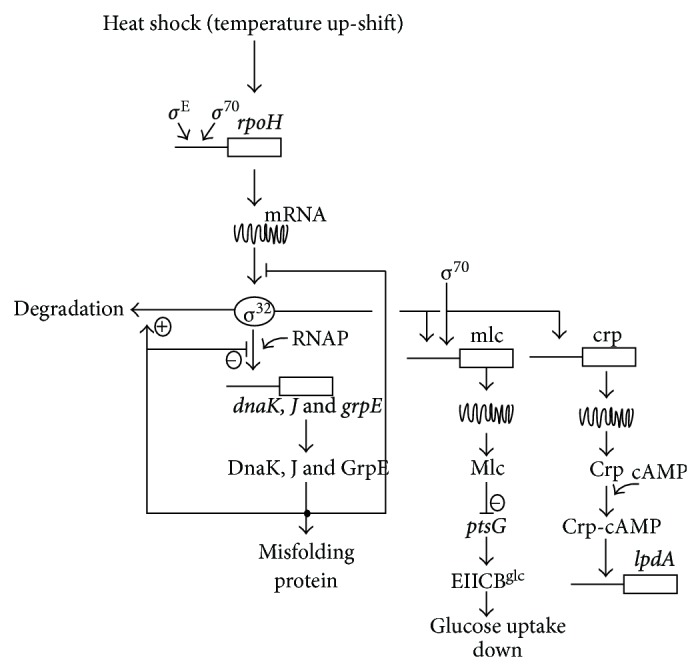
Effect of heat shock on gene and protein expressions and the fermentation characteristics.

The cyclic AMP (cAMP) receptor protein Crp activates transcription for more than 100 promoters. When bound to its allosteric effector cAMP, the Crp homodimer binds to the specific DNA sites near target promoters, enhancing the binding of RNA polymerase holoenzyme (RNAP) and facilitating the initiation of the transcription. It has been shown that *crp* gene expression increased, and *lpdA* gene expression followed the similar pattern upon heat shock. It was shown that *mlc* gene expression followed the same pattern as that of *rpoH* upon heat shock, which confirms that E*σ* ^32^ is involved in the expression of *mlc* gene. It has been shown that E*σ* ^32^ plays an important role in balancing the relative concentration of Mlc and EIICB in response to the availability of glucose in order to maintain inducibility of Mlc regulon at higher temperature [383]. When Mlc is overproduced it has been known to reduce acetate accumulation [384] and causes slow growth but gives better performance for recombinant protein production [385]. Mlc is a global regulator of carbohydrate metabolism, and regulates the expression of *pts* operon. Mlc represses *manXYZ *encoding enzyme II of the mannose PTS [386], *malT* encoding the activator of maltose operon, and *mlc* itself negatively [387]. Moreover, *ptsG* encoding enzyme IICB of the glucose PTS (EIICB^glc^) and the *pts* operon encoding general PTS proteins are also known to be repressed by Mlc [388, 389]. The *mlc* promoter is very weak because nucleotide sequence of −10 region of the promoter differs from the consensus sequence of the strong promoter of *E. coli*. In addition, Mlc expression is autoregulated by Mlc itself. Therefore, the intracellular concentration of Mlc is limited in *E. coli* [390]. The* mlc *gene has been known to be transcribed by two promoters, P1 and P2, and has a binding site of its own gene product. It has been shown by *in vitro* transcription assays of *mlc* gene that P2 promoter could be recognized by RNA polymerase containing the heat shock sigma factor *σ* ^32^ (E*σ* ^32^) as well as E*σ* ^70^, while P1 promoter is only recognized by E*σ* ^70^. The overall regulation mechanism against heat shock may be expressed as Figure 27 [374].

Let us consider the production mechanism of acetate at higher temperature. In the typical batch cultivation, the cells must switch efficiently from the rapid growth on a favored carbon source such as glucose to a much slower growth on the excreted by-products such as acetate. Acetate excretion occurs through the Pta-Ack pathway, or may possibly by Pox pathway. Acetate utilization occurs through AcCoA synthetase (Acs). This high-affinity acetate-scavenging enzyme converts acetate to AcCoA, where cells introduce it into the TCA cycle to generate energy and/or the glyoxylate pathway to build cell constituents. The higher expression of *acs* accelerates acetate assimilation in the presence of acetate [391, 392], which leads to the activation of glyoxylate pathway. Transcription occurs from two *σ* ^70^-dependent promoters such as the distal promoter *acs* P1 and proximal promoter *acs *P2 [391, 393]. While multiple factors influence transcription, Crp appears to function directly as the critical transcription factor. Cells control this acetate switch primarily by controlling the initiation of *acs* transcription from the major promoter *acs *P2 [391, 394]. Activation of *acs* transcription depends on cAMP-Crp. The cAMP-Crpbinds two sites within the *acs* regulatory region. However, it has been shown that Fis and Ihf independently modulate Crp-dependent activation of *acs *P2 transcription [395], and the mechanism is not so simple. As such, the activation of *crp* may cause *acs* to be upregulated. The *acs* gene is also under control of *rpoS*. It has been shown that *acs* is expressed in an *rpoS*-dependent manner during different phases of the growth [396].

Although cellular ATP may increase for short period after the temperature upshift in *E. coli* [397], it eventually decreases at higher temperature [353, 397]. It has also been reported that the specific CO_2_ production rate as well as O_2_ consumption rate increased upon temperature upshift [353, 397, 398]. As a result, the cell yield decreased and the cell maintenance increased [353, 399]. Although it has been reported that the TCA cycle flux increased upon temperature upshift at the specific growth rate of 0.08 h^−1^ [397], another investigation based on ^13^C-labeled experiment indicates that the TCA cycle flux became low at the dilution rates of 0.45 and 0.32 h^−1^ [400]. 

It has been reported that the respiration is activated during the temperature upshift [353]. It may be due to the activation of *cydB *gene expression. The *arcA *showed increased expression after the temperature upshift (especially first 30 mins) and modulated the expressions of such genes as *cydB, cyoA,* and *icdA*. The up-regulation of *arcA *gene may not be the direct effect of heat shock but indirectly due to lower dissolved oxygen concentration caused by the lower solubility at higher temperature [397]. 

It has been reported that superoxide dismutase gene (*sod*) is induced in response to the oxidative stress imposed by dioxygen or by the redox active compounds such as viologens or quinones caused by the temperature upshift [401]. It has also been reported that the exposure of *sodA/B* null mutant *E. coli* to aerobic heat stress caused a profound loss of viability [399, 402]. Moreover, the *sod* gene is under control of SoxRS, where it becomes significant under dual osmotic and heat stresses [403].

## 10. Fatty Acid Metabolic Regulation

 The acetate or fatty acid metabolism is also of practical interest. Here, consider this by looking at the effect of *fadR* gene knockout on the physiology of *E. coli*, where the parent and its *fadR* mutant are grown on glucose in a minimal medium under aerobic condition [299]. Compared to the parent strain, acetate production was reduced, whereas the biomass yield was enhanced in the *fadR* mutant. This cultivation phenomenon is similar to the result of Farmer and Liao [404]. The level of AceK, the bifunctional protein catalyzing phosphorylation/inactivation protein, was higher in the *fadR* mutant. Acetate-induced periplasmic transporter OppA showed lower expression level in the *fadR* mutant as compared to wild type [299]. Moreover, it was shown that Icl and MS, the two enzymes of the glyoxylate shunt, are significantly induced in the *fadR* mutant. CS, Acn, Fum, and MDH were coordinately upregulated to some extent. Moreover, NADP^+^-dependent malic enzyme (Mez) was upregulated whereas NAD^+^-dependent malic enzyme (Sfc) down-regulated in the *fadR* mutant as compared to wild type. In addition, Ppc showed lower activity in the *fadR* strain, whereas Pck activity increased. In the fermentative pathways, Ack activity is reduced in the *fadR* mutant as compared to the parent strain, which is in agreement with the decrease of acetate production in the *fadR* mutant. These trends indicate that the *fadR* mutant utilized the glyoxylate shunt for the replenishment of OAA for biosynthesis. The activation of the glyoxylate shunt bypassed the TCA cycle and thus prevented the loss of carbons as CO_2_ in ICDH and KGDH-catalyzed reactions.

Moreover, it was shown that PYR and AcCoA concentrations deceased, whereas the concentrations of isocitrate (ICIT), *α*KG, MAL, OAA, and aspartate (ASP) increased in the *fadR* mutant as compared to the parent strain [299]. These results are in agreement with those reported by van de Walle and Shiloach [405] who found that the operation of the glyoxylate shunt in *E. coli *BL21 resulted in accumulation of TCA cycle intermediates and higher biosynthesis fluxes. Similar to PYR, PEP concentration was also reduced in the *fadR* mutant. These variations reflected the action of the glyoxylate shunt in the *fadR* mutant. It is also observed that the intracellular concentrations of intermediates in the glycolysis and the PP pathway such as G6P and 6PG are reduced in the *fadR* mutant. Apart from these changes, the ratio of NADPH/NADP^+^ was lower, while that of NADH/NAD^+^ was higher in the* fadR* murtant as compared to wild-type strain.

**Figure 28:**
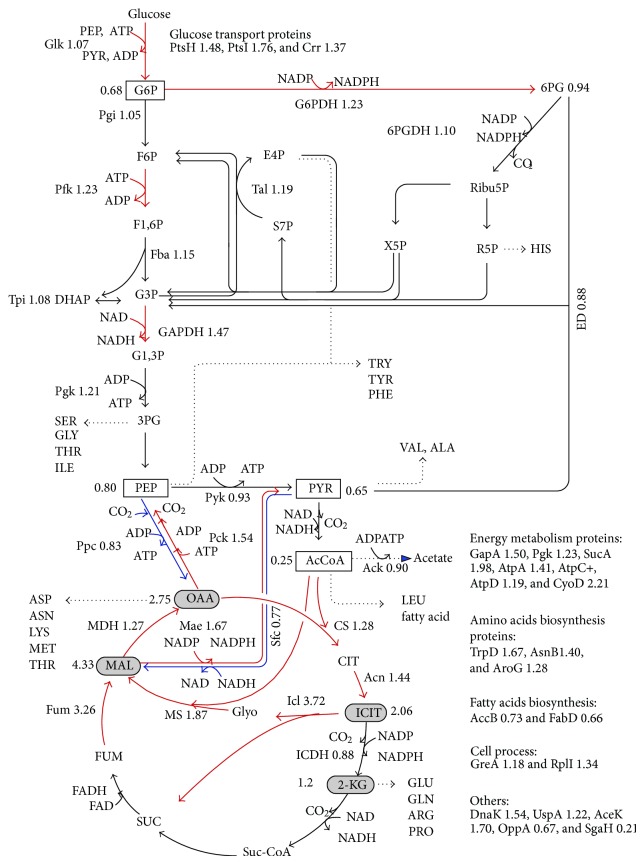
Metabolic pathways showing levels of enzymes (or proteins) and intracellular metabolite concentrations in the *fadR* mutant *E. coli* relative to those in the parent at the exponential phase grown in glucose minimal medium under aerobic condition. The numbers beside the protein names and the metabolites represent the ratios. Shaded and boxed metabolites mean increased and decreased intracellular concentrations in *fadR* mutant *E. coli*.

It has been reported that FadR is a transcriptional regulator with a Helix-turn-Helix motif, regulating metabolic pathways, such as the fatty acid biosynthesis and degradation, and glyoxylate shunt and playing a possible role in the regulation of amino acid biosynthesis directly or indirectly [406–408]. The overall effect of the *fadR* mutant is illustrated in Figure 28 [299].

Induction of the glyoxylate shunt led to the better utilization of AcCoA by increasing the carbon flow through this anaplerotic pathway, which is inferred from the significantly reduced intracellular concentration of AcCoA. The decrease of the intracellular AcCoA pool is therefore suggested to be responsible for the reduced acetate excretion in the *fadR* mutant. The pools of PEP and PYR are conjointly reduced in the *fadR* mutant due to the draining of carbon into the TCA cycle and the glyoxylate shunt. PEP is known to be a critical metabolite in *E. coli*. It involves not only in the PTS as a phosphoryl donor, but also in the regulation of many enzymes as an effecter [409]. It is, therefore, considered that the upregulations of the glycolytic enzymes, such as Pgi and Pfk in the *fadR* mutant are associated with the release from the inhibition due to the lower PEP concentration, since PEP is an inhibitor of both enzymes. Decrease of G6P concentration is responsible for the faster glucose uptake in the *fadR* mutant by the upregulation of PTS proteins, since G6P degrades the mRNA of PTS proteins by activating RNaseP enzyme [410]. Other glycolytic enzymes such as Fba, Tpi, GAPDH, and Pgk were concurrently upregulated in the *fadR* mutant to some extent to form more PEP and PYR, which are consistent with D'Alessio and Josse's results [411] that these enzymes are always regulated proportionally in *E. coli*. 

Concomitant with the induction of the glyoxylate shunt, some of the TCA cycle enzymes, such as CS, Acn, Fum, and MDH, were coordinately upregulated. Besides, SucA, a component of KGDH, and FumA showed higher expression levels. These upregulations are expected to fulfill the role in driving the increased carbon flux due to the action of the glyoxylate shunt. It is reported that CS and Acn, but not ICDH, are regulated in a coordinate mode, which may be due to the fact that citrate is an activator of Acn [412]. However, ICDH activity is subject to phosphorylation/inactivation control at the branch point of isocitrate to force the carbon flux towards the glyoxylate shunt, whose activity was slightly lower in the *fadR* mutant as compared to the parent strain. The phosphorylation/inactivation of ICDH is exerted by AceK, which is induced in the *fadR* mutant. Decreased carbon flux via ICDH, therefore, restricted the production of NADPH in the TCA cycle, as shown by the fact that the NADPH concentration is much lower in the mutant than in the parent strain. NADPH is an important cofactor for biosynthesis and mainly formed in the TCA cycle. Up to 60% of the total NADPH is produced in the TCA cycle in the parent strain under aerobic condition [189]. To meet the need for biosynthesis, NADPH has to be generated by other NADPH-producing pathways. One way is through NADPH-dependent malic enzyme, Mez, which is upregulated in the *fadR* mutant. The up-regulation of Mez is probably related to the reduced intracellular AcCoA concentration as this enzyme is repressed by glucose and AcCoA [413]. This up-regulation also played a role in supplying AcCoA from MAL via PYR in the *fadR* mutant. On the other hand, the activity of Sfc, which consumes NADH and produces NAD^+^ by coupling the conversion of PYR to MAL, reduced due to the smaller pool of PYR in the *fadR* mutant.

Proteome analysis demonstrated that the protein expressions in amino acids biosynthesis, such as AsnB, TrpD, and AroG F_1_F_2_ proton-translocating ATPase such as AtpA, AtpC, and AtpD and the ribosomal protein RplI as well as the transcription elongation factor GreA were upregulated in the *fadR* mutant. These genes are clustered and show growth rate dependent expression [414]. On the contrary, the levels of AccB and fabD involved in fatty acids biosynthetic pathways are negatively affected by *fadR *mutant, which is consistent with previous studies that the *E. coli* FadR functions as a repressor of the fatty acid degradative (*fad*) pathways and can also act as an activator of unsaturated fatty acid synthesis (*fab*) [405, 415, 416]. In addition, DnaK, the heat shock protein and UspA, the universal stress protein, are induced in the *fadR* mutant. These proteins are known to protect cells from stressful conditions such as heat shock, starvation, and stress stimulons; thus *fadR *mutation seems to be a stress to the *E. coli* cell. The *uspA* is a member of the *fadR* regulon, and the transcription of *uspA *is derepressed during exponential growth in *fadR* null mutants [416]. Previous studies revealed that RpoS-regulated genes, periplasmic transporters for amino acids and peptides, and metabolic enzymes are induced either by acetate or at low pH [417, 418]. Of these, it is considered that the downregulation of OppA is related to the less accumulation of acetate in the *fadR* mutant compared to the parent strain.

## 11. Response to Nutrient Starvation and the Metabolic Regulation by RpoS

Although many industrial fermentations are conducted in the batch mode, most of the studies have focused on the cell growth phase, and little attention has been paid to the late growth and stationary phases. Since the important metabolites are produced at the early stationary or stationary phases, it is quite important to clarify the metabolic regulation that occurs during these phases. During batch fermentation, the cultural conditions change from glucose rich to acetate rich condition, and change further to the carbon-starved condition in *E. coli*. Several global regulators such as RpoD, SoxRS, Cra, FadR, and IclR may help *E. coli *to cope with different kinds of metabolic stresses. Apart from these regulators, RpoS, the master regulator of the stationary phase or stress-induced genes in *E. coli *regulates such genes as those for the carbohydrate PTS, *crr*, glycolytic pathway genes such as *fbaB *and *pfkB*, the acetate-forming gene *poxB*, the nonoxidative PP pathway genes such as *talA *and *tktB*, and TCAcycle genes such as *acnA *and *fumC*. In addition, some of the amino acid and fatty acid metabolic pathway genes such as a*rgH*, *aroM, *and *yhgY* and energy metabolism genes such as *narY*, *appB,* and* ldcC *have also been reported to be regulated in an *rpoS*-dependent manner [419–425]. 

*E. coli* cells are exposed to different stress conditions such as oxidative stress, acid stress or stresses from particular ion or carbon limitation at different phases of growth. Fortunately, *E. coli* cells possess several regulatory proteins, which through the regulation of a large number of genes help bacteria to cope with continuously changing environment under different stress conditions, including acid stress, or other stresses mentioned above [164]. Of these stress conditions, acid stress, particularly stress from acetate gives a problem for the growth in *E. coli*. In addition, acetic acid has been recognized to be a problem in recombinant protein production as it easily passes through the thin lipid layer of the bacterial cell wall and cause damage to the protein production [419, 426]. It has been reported that the stress regulatory protein RpoS regulates the expression of approximately 78 genes in *E. coli* during acid stress [419, 427]. 

In general, the bacterial culture medium is considered to be rich in carbohydrate such as glucose as the sole carbon source during the exponential growth phase. As the cell such as *E. coli *utilizes the glucose, acetate is produced as the major fermentative product under aerobic condition, and the cell exhibits a diauxic shift which causes a termination of the exponential growth phase and transition towards the stationary phase. Then, *E. coli* utilizes acetate as a carbon source during early stationary phase of growth. When acetate is used up, *E. coli* starts to utilize amino acids as carbon and nitrogen sources during stationary phase. The complex changes occurring among the major metabolic pathway enzymes, their respective genes and the intermediary metabolites, during a shift from carbon rich to carbon-limited conditions, have been a major topic of interest in the metabolic regulation analyses. RpoS is a global regulator that regulates the expressions of many genes at the onset of stationary phase or carbon-starved conditions and other stress conditions in *E. coli* [420, 423, 424]. 

RpoS is a sigma factor of RNA polymerase. It is known that the core RNA polymerase consists of four subunits such as two *α*, one *β* and one *β*′. Part of the RNA polymerase that recognizes the promoter-binding site is generally known as sigma factor (*σ*). Without the sigma factor, RNA polymerase remains inactive [428]. *E. coli* possesses seven different *σ* factors [428] as mentioned before. Depending on the environmental condition, different sigma factors bind with the RNA polymerase so that particular gene expressions are initiated. Of these seven different sigma factors, *rpoS* or *σ* ^38^ is important in bacterial metabolism as this transcription factor has been shown to be associated with different kinds of stresses in *E. coli* [420, 421, 428]. Unlike other regulators, expressions of which are stimulated by certain effector molecules, and these regulators then function by binding to the promoter sites of particular genes, where RpoS itself is a transcription factor, and regulates the expressions of genes at the transcriptional levels. However, once the transcription starts, the sigma factor dissociates from the RNA polymerase. 

RpoS has been shown to stimulate the expressions of several oxidative stress response genes such as *katE*, *katG*, *sodC*, and *dps* and osmotic stress response genes such as *osmE*, and *osmY*. Strains lacking a functional *rpoS* gene also failed to express the genes for acid resistance such as *gadA* and *gadB*, near-UV resistance gene *nuv*, acid phosphatase genes *appAR*, and so forth [420, 421]. The intracellular level of RpoS itself is regulated by various mechanisms depending on the stress type and growth conditions. For example, *rpoS* transcription is stimulated by a reduction in growth rate, whereas translation is stimulated by osmotic shock, low temperature, or pH downshifts [420, 421, 427]. The third mechanism that controls the RpoS level is through proteolysis. While under normal situation, RpoS is rapidly degraded by ClpXP proteases, and the proteolytic activity of this enzyme is considerably reduced under stress conditions [420, 429, 430]. 

Although the roles of RpoS are originally described for various types of stress response, it has been demonstrated that the regulatory roles of *rpoS* are not restricted to the stress response genes only. In *E. coli*, RpoS-dependent genes are found all over the chromosome, whose function ranges from DNA repair and protein synthesis to the transport, biosynthesis and metabolism of sugars, amino acids, and fatty acids. Notably, RpoS is found to regulate the expression of DNA repair enzymes such as the exonuclease encoded by *xth*A and the methyl transferase encoded by *ada*, the gene that determines the cell morphology such as *bol*A, the genes encoding transport, and binding proteins such as *gabP*, and *ugpEC*. In addition, a considerably large number of unknown proteins are invariably affected by *rpoS* mutation [422, 427]. Considering the wide range of activities of RpoS, it seems obvious that RpoS could have significant contribution in the maintenance of *E. coli* metabolic pathways at the stationary phase under carbon-starved conditions. 

The complexity of the metabolic system of *E. coli* is exemplified by the fact that many metabolic pathway genes are found to be regulated by more than one global regulator. For example, *icdA* of the TCA cycle is regulated by RpoD and Cra, *acnA* and *fumC* of the TCA cycle are regulated by SoxRS and RpoS, *aceA* and *aceB* of the glyoxylate pathways are regulated by Cra and IclR, and so forth [319, 431–435]. Moreover, the metabolic pathway of *E. coli* consists of several genes that possess isogenes. These iso-genes are known to encode backup enzymes in response to certain environmental stimuli, and the expressions of these enzymes are often regulated by one or more of the global regulators. It appears that the stress inducible metabolic pathway genes constitute the functional units through which different global regulators coordinate metabolic activities in the face of changing culture environment. 

In *E. coli,* transketolase is encoded by *tktA* and *tktB* genes, and transaldolase is encoded by *talA* and *talB* genes, respectively. TktA and TalB are reported to be the major enzymes to catalyze transketolase and transaldolase reactions, and TktB and TalA are the minor enzymes of the nonoxidative PP pathway [436, 437]. The non-oxidative PP pathway is important as E4P, the important precursor of the aromatic amino acids, and as S7P, which is the cell wall components of *E. coli,* are synthesized only through the non-oxidative branch. E4P and S7P are produced through the consecutive reactions catalyzed by Tkt and Tal [436, 438]. While the physiological roles of the major and minor enzymes have been elucidated, the reports on the positive regulation of the minor transketolase (encoded by *tktB*) and transaldolase (encoded by *talA*) of the non-oxidative PP pathway by the stress regulator RpoS indicates that these genes might play significant role at the carbon-starved conditions [422, 427, 439]. 

On the other hand, the TCA cycle genes such as *fumC* and *acnA* encode FumC and AcnA enzymes of the TCA cycle, respectively. While fumarase catalyzes the conversion of FUM to MAL by the hydration reaction, Acn catalyzes the conversion of CIT to *cis*-aconitate to ICIT through dehydration and hydration reactions [440]. However, both FumC and AcnA have iso-enzymes such as FumA and AcnB, which are encoded by the genes *fumA* and *acnB*, respectively. It has been reported that FumA and AcnB play the major roles of fumarase and aconitase when the cell grows under optimum growth condition. Both FumA and AcnB possess Fe-S clusters that make these enzymes vulnerable to oxidative stress, and under such condition, FumC and AcnA play as back-up enzymes [432, 434, 435, 441]. It has been reported that RpoS regulates the expressions of *fumC* and *acnA* at the stationary phase of growth. Note that it is also reported that the expressions of both *fumC* and *acnA* genes are also regulated by the oxidative stress regulators SoxRS as well [285, 442]. In summary, RpoS plays important roles at the late growth phase and the stationary phase [195, 396].

In order to determine how the cell cope with the absence of a vital gene like *rpoS*, it may be identified such genes that were upregulated at the early stationary phase from the microarray data [425]. A total of 208 genes were upregulated in the mutant excluding the genes for hypothetical proteins. Among these genes, 25 genes (~12%) were upregulated in both phases of growth. Microarray data revealed that, of the central metabolic pathways, significant reduction of the expression was observed for several genes during early stationary phase, with the exception of *fumC*, which was upregulated. The down-regulation of several genes such as *glgA* and *glgS* involved in glycogen synthesis is also an indicator of the bacterial adaptive response to carbon-limited conditions in the absence of *rpoS* background. 

As mentioned earlier, apart from the TCA cycle activity, accumulation of acetate throughout the cultivation period was another notable feature of the *rpoS* mutant [425]. While two genes, *ackA* and *poxB*, involved in acetate production are down-regulated in the mutant at the early stationary phase, microarray data [425] indicate that acetate production could be stimulated by the up-regulation of L-cysteine biosynthesis genes such as *cysD*, *cysE,* and *cysK*, catalyzing the reactions that generate acetate as a by-product [164]. Moreover, enzymes involved in the acetate catabolism such as AcCoA synthetase encoded by* acs*, the glyoxylate shunt enzymes encoded by the *aceBAK* operon, and the TCA cycle genes such as *gltA*, *mdh*, and *sdhC* were down-regulated during the stationary phase [424, 443, 444]. 

Down-regulation of *acs* during the early stationary phase resulted in a decrease in the intracellular pool of AcCoA in the mutant compared to the wild-type strain. While the major route for AcCoA formation was less expressed, the other pathways for AcCoA formation rely on fatty acid degradation pathway [164]. Several genes that participate in *β*-oxidation of fatty acids, particularly *fadA* and* fadB* are significantly upregulated, and the fatty acid biosynthesis genes such as *accB*, *accC*, *accD, *and *fabF* were also upregulated in the mutant [425]. The expression of the fatty acid degradation regulator, *fadR,* was significantly high at the early stationary phase of growth. It is known that FadR regulates fatty acid metabolism by binding to the DNA that contains *fadB *promoter binding sites, and in this way *fadR* controls fatty acid metabolism [445, 446]. Down-regulations of *ace*A and *ace*B genes correspond to the higher expression of *fadR*, where FadR represses the glyoxylate shunt encoded by *aceBAK* by directly regulating the activation of the glyoxylate shunt repressor, *iclR* [405, 447]. The higher expressions of *fadR* and *iclR *also caused acetate accumulation.

## 12. Conclusions

It was shown in the present paper that a variety of regulation mechanisms are present in response to the changes in culture environment, where the appropriate global regulators or transcription factors together with sigma factors play important roles. Although the findings on the metabolic regulation mechanisms so far are only a tip of ice verg, it is a grand challenge to uncover the overall regulation mechanism in particular by systems biology approach to understand the mystery of how and why the living organisms are so well and efficiently organized for survival.

## Figures and Tables

**Table 1:** Sigma factors and their functions.

*σ* ^ 19^	Ion transport
*σ* ^ 24^	Extreme temperature
*σ* ^ 28^	Flagella genes
*σ* ^ 32^	Heat shock
*σ* ^ 38^	Stationary phase or carbon starvation, and so forth
*σ* ^ 54^	Nitrogen regulation
*σ* ^ 70^	House keeping

**Table 2:** Effect of dilution rate on fermentation characteristics of wild type *E. coli*.

Dilution rate (h^−1^)	Biomass conc. (g/L)	Specific glucose uptake rate (mmol/g/h)	Specific acetate formation rate (mmol/g/h)	Biomass yield (g/g)	Specific CER (mmol/g/h)
0.2	1.45 ± 0.06	3.07 ± 0.13	ND∗	0.37 ± 0.015	9.15
0.4	1.87 ± 0.09	4.75 ± 0.23	0.01	0.47 ± 0.023	11.61
0.6	2.0 ± 0.09	6.67 ± 0.3	0.88 ± 0.04	0.5 ± 0.023	13.17
0.7	1.93 ± 0.08	8.05 ± 0.34	1.33 ± 0.06	0.48 ± 0.02	15.83

∗ND: not detectable, where glucose detectable limit is 0.038 g/L.

**Table 3:** Regulators involved in regulating glutamate-dependent acid resistance.

Protein	Descriptor	Function in acid resistance
RpoD	*σ* ^ 70^	Transcription of *gadA/BC *
RpoS	*σ* ^ 38^	Transcription of *gadX *
EvgAS	Two-component signal transduction	Activates *ydeO* and* gadE* transcription
YdeO	AraC-like regulator	Activates *gadE* transcription
GadE	LuxR-related activator	Required for acid resistance, binds to Gad box, activates transcription of *gadA/BC*, autoactivates *gadE*, and represses *ydeO *
GadX	AraC-like regulator	Activates *gadE*, coactivates *gadA/BC,* and represses *gadW *
GadW	AraC-like regulator	Inhibits RpoS production, activates *gadE*, can coactivate *gadA/BC* at pH 8
Crp	cAMP receptor protein	Inhibits RpoS production
TrmE	Era-like GTPase	Activates *gadE* mRNA production and stimulates translation of *gadA *and *gadB* mRNA
HNS	Histone-like protein	Negative regulator
TorR	Response regulator of TMAO reductase	Negative regulator of *gadE *

[333].
